# Sitagliptin attenuates arterial calcification by downregulating oxidative stress-induced receptor for advanced glycation end products in LDLR knockout mice

**DOI:** 10.1038/s41598-021-97361-w

**Published:** 2021-09-08

**Authors:** Chih-Pei Lin, Po-Hsun Huang, Chi-Yu Chen, Meng-Yu Wu, Jia-Shiong Chen, Jaw-Wen Chen, Shing-Jong Lin

**Affiliations:** 1 Department of Laboratory Medicine, Taipei Tzu Chi Hospital, Buddhist Tzu Chi Medical Foundation, New Taipei City, Taiwan; 2 Division of Clinical Pathology, Taipei Tzu Chi Hospital, Buddhist Tzu Chi Medical Foundation, New Taipei City, Taiwan; 3 Department of Laboratory Medicine and Biotechnology, College of Medicine, Tzu Chi University, Hualien, Taiwan; 4 Department of Critical Medicine, Taipei Veterans General Hospital, Taipei, Taiwan; 5 Cardiovascular Research Center, National Yang Ming Chiao Tung University, Taipei, Taiwan; 6 Institute of Clinical Medicine, National Yang Ming Chiao Tung University, Taipei, Taiwan; 7 Department of Emergency Medicine, Taipei Tzu Chi Hospital, Buddhist Tzu Chi Medical Foundation, New Taipei City, Taiwan; 8 Department of Emergency Medicine, School of Medicine, Tzu Chi University, Hualien, Taiwan; 9 Division of Cardiology & Healthcare and Management Center, Taipei Veterans General Hospital, Taipei, Taiwan; 10 Institute of Pharmacology, National Yang Ming Chiao Tung University, Taipei, Taiwan; 11 Division of Cardiology & Department of Medical Research, Taipei Veterans General Hospital, Taipei, Taiwan; 12 Taipei Heart Institute, Taipei Medical University, Taipei, Taiwan; 13 Division of Cardiology, Heart Center, Cheng-Hsin General Hospital, Taipei, Taiwan; 14 Division of Clinical Pathology & Department of Laboratory Medicine, Taipei Tzu Chi Hospital, Buddhist Tzu Chi Medical Foundation, 112, No. 201, Sec. 2, Shih-Pai Road, Taipei, Taiwan

**Keywords:** Cardiovascular diseases, Calcification

## Abstract

Diabetes is a complex disease characterized by hyperglycemia, dyslipidemia, and insulin resistance. Plasma advanced glycation end products (AGEs) activated the receptor for advanced glycation end products (RAGE) and the activation of RAGE is implicated to be the pathogenesis of type 2 diabetic mellitus (T2DM) patient vascular complications. Sitagliptin, a dipeptidyl peptidase-4 (DPP4) inhibitor, is a new oral hypoglycemic agent for the treatment of T2DM. However, the beneficial effects on vascular calcification remain unclear. In this study, we used a high-fat diet (HFD)-fed low-density lipoprotein receptor deficiency (LDLR^−/−^) mice model to investigate the potential effects of sitagliptin on HFD-induced arterial calcification. Mice were randomly divided into 3 groups: (1) normal diet group, (2) HFD group and (3) HFD + sitagliptin group. After 24 weeks treatment, we collected the blood for chemistry parameters and DPP4 activity measurement, and harvested the aorta to evaluate calcification using immunohistochemistry and calcium content. To determine the effects of sitagliptin, tumor necrosis factor (TNF)-α combined with S100A12 was used to induce oxidative stress, activation of nicotinamide adenine dinucleotide phosphate (NADPH), up-regulation of bone markers and RAGE expression, and cell calcium deposition on human aortic smooth muscle cells (HASMCs). We found that sitagliptin effectively blunted the HFD-induced artery calcification and significantly lowered the levels of fasting serum glucose, triglyceride (TG), nitrotyrosine and TNF-α, decreased the calcium deposits, and reduced arterial calcification. In an *in-vitro* study, both S100A12 and TNF-α stimulated RAGE expression and cellular calcium deposits in HASMCs. The potency of S100A12 on HASMCs was amplified by the presence of TNF-α. Sitagliptin and Apocynin (APO), an NADPH oxidase inhibitor, inhibited the TNF-α + S100A12-induced NADPH oxidase and nuclear factor (NF)-κB activation, cellular oxidative stress, RAGE expression, osteo transcription factors expression and calcium deposition. In addition, treatment with sitagliptin, knockdown of RAGE or TNF-α receptor blunted the TNF-α + S100A12-induced RAGE expression. Our findings suggest that sitagliptin may suppress the initiation and progression of arterial calcification by inhibiting the activation of NADPH oxidase and NF-κB, followed by decreasing the expression of RAGE.

## Introduction

Type 2 diabetes mellitus (T2DM) patients have a higher risk of developing atherosclerosis, artery calcification, morbidity and mortality rates than the non-diabetic population^1,2^. Although the mechanisms causing T2DM patients to have a higher risk of vascular calcification than the non-diabetic population are not yet fully understood, it is believed that the hyperglycemia and hyperlipidemia of T2DM patients induce a cascade of events leading to arterial calcification^3^. Vascular smooth muscle cells (VSMCs), are believed to be involved in the pathogenesis of vascular calcification^4^. Medial calcification represents a concentric calcification that is preceded by matrix vesicle-nucleated mineralization accompanied by calcium phosphate deposits in the arterial tunica media^5^. Important transcription factors, such as Msh homeobox 2 (MSX2), Osterix, Bone matrix protein-2 (BMP-2) and runt-related transcription factor 2 (RUNX2), are crucial in the programing of osteogenesis^6,7^. MSX2 and RUNX2 were highly correlated with arterial calcification. The initiation of a high-fat diet-induced low-density lipoprotein receptor knockout (LDLR^−/−^) mice model demonstrated the pattern of MSX2 expression in cardiac valves and adventitia myofibroblasts^8^. MSX2 was a BMP-2-inducible transcription factor that inhibited osteoblast terminal differentiation in early osteoblast development^9^. Expression of MSX2 promoted osteogenic differentiation and suppressed adipogenesis via paracrine signals^10^. Osteo/chondrogenic transcription factors including SOX9, RUNX2 and MSX2 were upregulated in the arterial calcification^11^. In previous studies, MSX2-Wnt signaling participated in the aortic valve and medial calcification in high-fat diet-supplement LDLR^−/−^ mice^10,12,13^. Moreover, TNF-α regulated the osteogenic signals and participated in aortic calcification in LDLR^−/−^ mice. The MSX2-Wnt signaling cascades were involved in TNF-α signal in high-fat diet-induced obesity and T2DM^10,14^. Understanding the association between diabetes complications and VSMCs osteotransformation may aid the development of anti-arterial calcification therapeutic for T2DM patients^13,15,16^.

Diabetic patients were found to have a higher level of plasma advanced glycation end products (AGEs), tumor necrosis factor (TNF)-α, and receptors for AGEs (RAGEs) than the nondiabetic population^16,17^. The RAGE was expressed in the endothelial and smooth muscle cells, and the activation of RAGE augmented inflammation by stimulating the release of inflammatory cytokines and generation of oxidative stress^18^. AGE/RAGE signaling was reported to induce VSMCs calcification by activating nicotinamide adenine dinucleotide phosphate (NADPH) oxidase (Nox) 1 pathway. Activated NAPHD-induced superoxide and hydrogen peroxide led to activation of inflammation and increased the expression of RAGE^19^. It is suggested that the activation of nuclear factor (NF)-κB led to activation of RAGE, which led to a wide spectrum of pathological inflammation conditions and various diseases such as atherosclerosis^20^. The activation of NF-κB was reported to upregulate RAGE expression and this positive feedback loop between RAGE and NF-κB signaling pathway results in a perpetuation of inflammation stats^21^. This RAGE/NF-κB interaction theory explained why oxidative stress caused by ureteral obstruction only caused severe arterial calcification in transgenic S100A12 C57BL/6J mice but not the wild-type animals^19^. Recent studies suggested that T2DM patients have a higher level of circulatory S100A12 than non-diabetic people^22^. S100A12 is a potent agonist for RAGEs. The S100A12 transgenic apolipoprotein-E (ApoE) null mice on regular rodent chow developed much more server calcified atherosclerotic plaques and medial calcification than the wild-type ApoE null mice^23^. RAGE-S100A12 signaling is reported to cause coronary artery diseases and vascular calcification^23,24^.

Dipeptidyl peptidase-4 (DPP4) is a multifunctional enzyme found in catalytically active soluble form in plasma and on the surface of most cell types^25^. DPP4 knockout mice study revealed that the absence of this enzyme improved glycemic control and reduced animal fat mass^26^. DPP4 degraded incretin hormones, such as type I glucagon-like peptide (GLP-1) that is widely known for its regulatory effect in glucose metabolism^27^. DPP4 inhibitors were a new class of oral anti-hyperglycemic medications used for the treatment of T2DM without causing weight gain^28^. Sitagliptin, an absorbable DPP4 inhibitor, was found to retard the progression of carotid atherosclerosis in T2DM patients^29,30^ and ApoE-deficient mice^31,32^ by improving endothelial function and imparting anti-inflammatory effects. Recent animal studies have suggested that the DPP4 inhibitor may contribute to its anti-atherosclerotic effects by reducing the reactive oxygen species (ROS) generation, preventing mitochondrial depolarization, improving endothelial functions, and reducing vascular inflammation^33^. It has been reported that TNF-α plays a crucial role in arterial calcification in diabetic LDLR^−/−^ mice^13^. Our previous study showed that TNF-α and vascular tumor necrosis factor receptor (TNFR) signaling lead to human aortic smooth muscle cells (HASMCs) calcification and antioxidants blunt the TNF-α/TNFR signaling to retard the HASMCs calcification^34^.

To our knowledge, no one has investigated the effect of sitagliptin on the progression of T2DM patients’ arterial calcification. In this study, we hypothesized that sitagliptin improved arterial calcification. We used a chow or high-fat diet (HFD)-fed LDLR^−/−^ mice supplemented with vehicle or Sitagliptin to investigate the effect on animal arterial calcification and HASMCs model to determine how sitagliptin exerts its effect. We demonstrated that sitagliptin attenuated the levels of weight, aortic calcium content, DPP4 activity and arterial calcification in HFD-induced LDLR^−/−^ mice. Medial calcification is mostly associated with hyperglycemia^15^. We used HASMCs in the *in-vitro* study to mimic VSMCs. The results showed that TNF-α and S100A12 increased the levels of RAGE expression and calcium deposition. Treatment with sitagliptin decreased the TNF-α-induced osteogenic gene expression including RAGE, RUNX2, MSX2 and BMP-2. Moreover, sitagliptin reduced the activity of NADPH oxidase by inhibiting the expression of p47, thereby reducing the production of superoxide and hydrogen peroxide and the activity of NF-κB.

## Results

### Effects of HFD and sitagliptin on HFD-induced LDLR^−/−^ body weight gain, aortic calcification and atherosclerotic plaque formation

**Figure 1:**
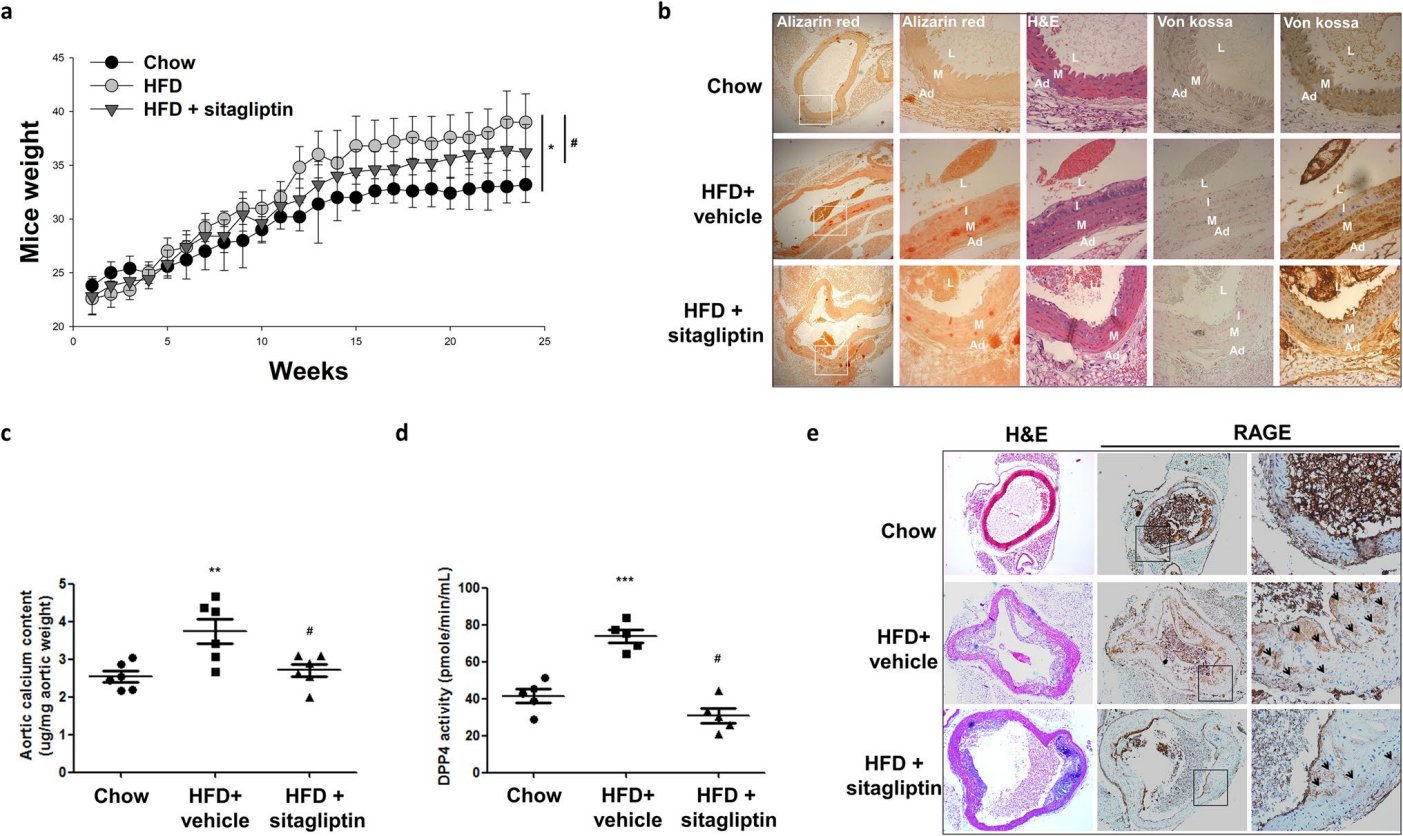
HFD promoted calcification, which was attenuated by sitagliptin, in male LDLR^−/−^ mice. LDLR^−/−^ mice were fed with a chow diet or HFD and simultaneously treated with sitagliptin (Sita, 100 mg/kg/day) for 6 months. **(a)** LDLR^−/−^ mouse body weight was recorded weekly. **(b)** HFD-induced artery atherosclerotic plaque formation was detected by H&E staining. HFD-induced artery calcification was detected by alizarin red staining. HFD promoted aortic calcification, which was dominant in the medial layer. The arrows point to the area of calcium deposition. The lumen showed blood cell remnants. *L* lumen, *I* intima, *M* media, and *A* adventitia. (ND = 6, HFD = 6, and sitagliptin = 6). LDLR^−/−^ mice aorta were photographed at × 100 and × 400 magnifications. **(c)** Aortic calcium levels and **(d)** DPP4 activity were assayed. **(e)** Immunostaining of aortic RAGE showed that sitagliptin could significantly decrease the stimulatory effects of HFD on RAGE. Arrowhead indicates positive RAGE position. *P < 0.05 compared with the chow group, and ^#^*P* < 0.05 compared with the HFD groups (Aortic calcium levels; Chow = 6, HFD + vehicle = 6, and HFD + sitagliptin = 6. DPP4 activity was assayed; Chow = 5, HFD + vehicle = 5, and HFD + sitagliptin = 5).

We found that the HFD-fed LDLR^−/−^ mice had a significantly higher body weight than the regular chow-fed mice (Fig. 1a; 33.8 ± 1.9 vs. 41.8 ± 2.3 g). Sitagliptin attenuated HFD-induced body weight gain (Fig. 1a; 41.8 ± 2.3 vs. 36.8 ± 2.5 g). HFD-fed mice had a higher level of blood glucose, triglyceride (TG), cholesterol (CHL), low-density lipoproteins (LDL) and TNF-α than the regular chow-fed mice (Table 1). These findings suggest that HFD fed LDLR^−/−^ mouse is a valid model as an obesity-associated T2DM animal model. HFD-fed LDLR^−/−^ mice treated with sitagliptin drastically lowered the fasting TG (539 ± 121 vs. 358 ± 128 mg dL^−1^), TNF-α levels (53 ± 17 vs. 30 ± 7 pg mL^−1^) and the levels of blood glucose. Numerically, a moderate reduction in LDL was observed, but the level of reduction was not statistically significant.

**Table 1:** Metabolic parameters after 24 weeks of dietary challenge and sitagliptin (100 mg/kg/day) treatment.

	Chow (n = 6)	HFD + vehicle (n = 6)	HFD + sitagliptin (n = 6)
Calcium (mg dL^−1^)	8.8 ± 0.6	8.8 ± 0.2	9.1 ± 0.1
PHOS (mg dL^−1^)	8 ± 1	7.5 ± 0.2	7.0 ± 1.0
Glucose (mg dL^−1^)	105 ± 15	274 ± 31*	223 ± 22^#^
CHOL (mg dL^−1^)	255 ± 42	1098 ± 273*	1105 ± 135
HDL (mg dL^−1^)	60 ± 22	65.0 ± 15.0*	65.0 ± 2.0
LDL (mg dL^−1^)	91 ± 27	566 ± 166*	463 ± 68
TG (mg dL^−1^)	117 ± 32	539 ± 121*	358 ± 128^#^
TNF-α (pg mL^−1^)	10.8 ± 6.4	55.3 ± 17.5*	30.5 ± 7.7^#^
Nitrotyrosin (ug mL^−1^)	9.9 ± 4.6	34.8 ± 2.6*	10.5 ± 4.5^#^

Male LDLR^−/−^ mice (n = 6 per group) were maintained on either mouse chow or HFD for 24 weeks and treated with either vehicle or 100 mg^−1^ kg^−1^ day^−1^ sitagliptin in vehicle. Data is presented as the mean ± SD.

*PHOS* phosphate, *CHOL* cholesterol, *HDL* high-density lipoprotein cholesterol, *LDL* low-density lipoprotein cholesterol, *TG* triglyceride.

*P < 0.05 vs. chow.

^#^*P* < 0.05 vs. HFD.

HFD-fed LDLR^−/−^ mice had a remarkable increase in calcification in the medial layer of the upper descending aorta as indicated by alizarin red staining and aorta calcium content analysis (Fig. 1b,c) compared to the regular chow-fed mice. HFD also caused the LDLR^−/−^ mice to have much higher levels of serum DPP4 activity (Fig. 1d). Sitagliptin treatment significantly blunted the HFD-induced calcium deposition in the media of the descending aorta (Fig. 1b), aortic acid-extracted calcium content (Fig. 1c) and the levels of serum DPP4 activity (Fig. 1d). In addition, we found that HFD-fed LDLR^−/−^ mice had a higher level of RAGE in the medial layer of the upper descending aorta (Fig. 1e). Orally administration of sitagliptin drastically reduced the expression of RAGE in aorta (Fig. 1e).

### Effects of TNF-α and S100A12 on RAGE expression and calcium deposition in HASMCs

**Figure 2:**
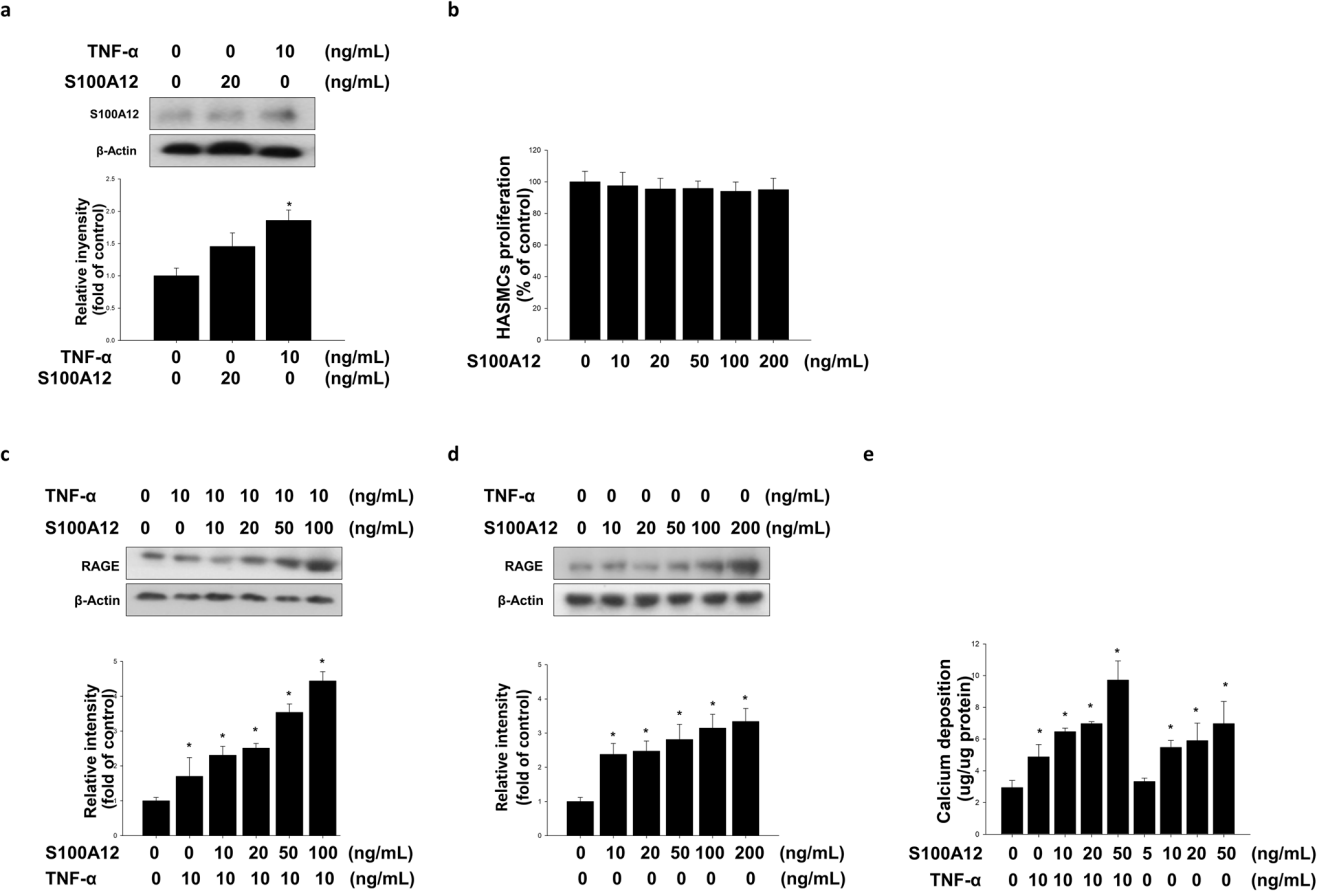
Effect of the combination of TNF-α with S100A12 on induced RAGE accumulation and calcium deposition in HASMCs. **(a)** The accumulation of S100A12 by TNF-α but not recombinant S100A12 in HASMCs was assayed. **(b)** The cytotoxicity effect of S100A12 was determined by MTT assay. The induction of RAGE by **(c)** S100A12 combined with TNF-α or **(d)** S100A12 alone in HASMCs was determined by Western blotting assay. The accumulation of RAGE by combination of S100A12 with TNF-α in HASMCs was assayed. S100A12 enhanced TNF-α-induced RAGE protein accumulation in HASMCs. **(e)** HASMCs were cultured in osteogenic differentiation medium treatment with S100A12 for 4 days in the presence or absence of TNF-α. Calcium deposition was induced dose-dependent by TNF-α for 4 days. N = 6 for each set of experiments. *P < 0.05 compared with the control group.

T2DM patients had higher levels of TNF-α and S100A12 compared to non-diabetic subjects^35^ and was associated with the severity of coronary artery diseases and vascular calcification^23,24^. We use the HASMC model to investigate (1) whether TNF-α + S100A12 induced HASMC calcification and (2) and how sitagliptin blunted the effects of TNF-α + S100A12 treatment. TNF-α (10 ng mL^−1^) treatment significantly increased the expression of S100A12 in HASMCs but did not significantly stimulate the expression of S100A12 with recombinant S100A12. (Fig. 2a). We demonstrated the expression of RAGE with S100A12 treatment in the presence or absence of TNF-α. S100A12 did not impair the cell viability of HASMCs (Fig. 2b). S100A12 produced a dose dependent increase on the expression of RAGE and the potency of S100A12 on RAGE expression appeared to be amplified by the presence of TNF-α (Fig. 2c,d). The calcium deposits on S100A12-treated HASMCs were much more pronounced in the presence of TNF-α (Fig. 2e).

### Sitagliptin, N-acetyl cysteine (NAC), TNFR-1 small interfering RNA (siRNA) and RAGE siRNA affected the expression levels of RAGE and calcium deposits on TNF-α/S100A12-treated HASMCs

**Figure 3:**
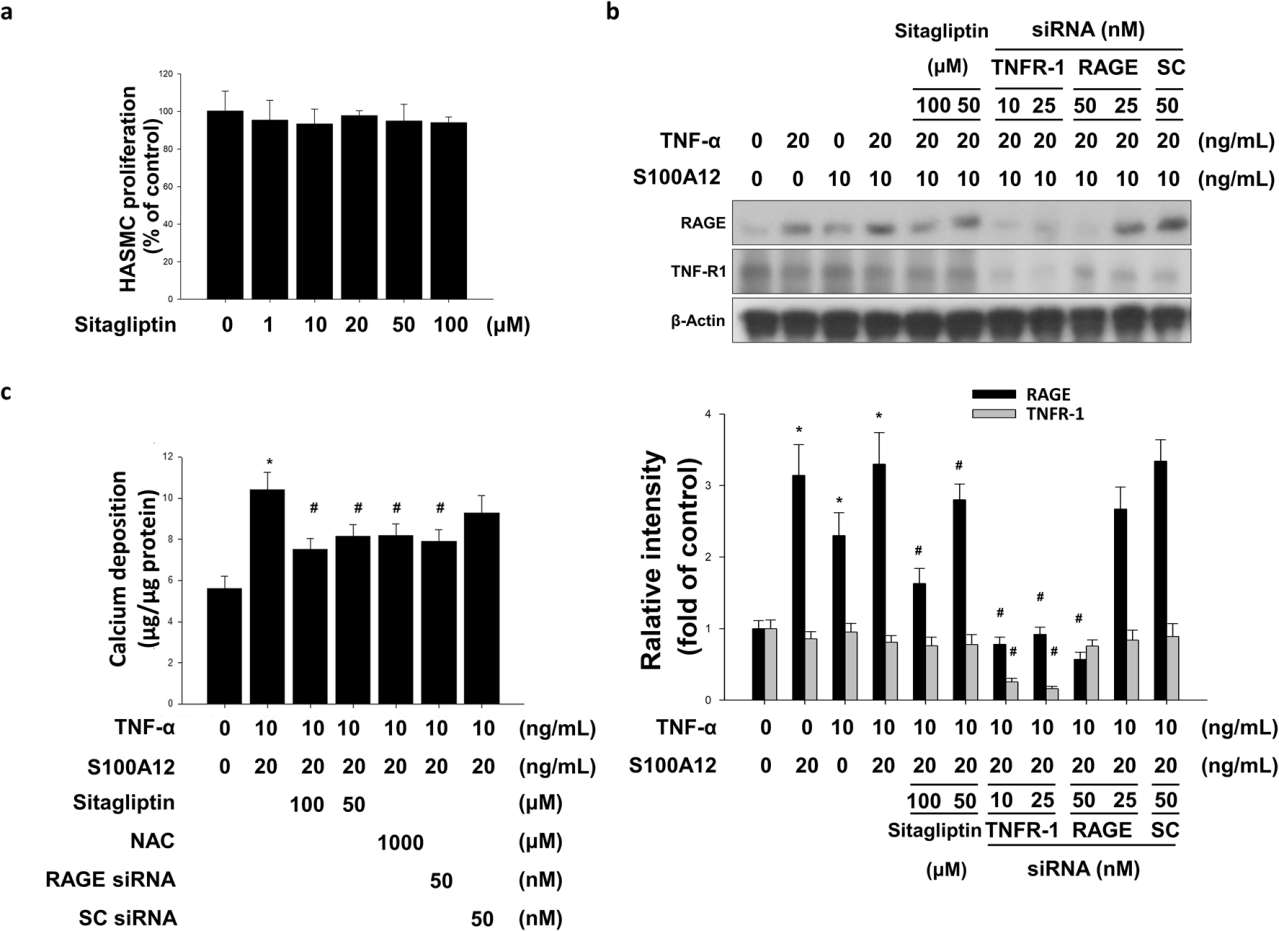
Sitagliptin attenuated TNF-α combination with S100A12-induced calcium deposition in HASMCs. **(a)** The cytotoxicity effect of sitagliptin was determined by MTT assay. **(b)** Sitagliptin and NAC attenuated the calcium deposition mediated by TNF-α combined with S100A12. The induction of RAGE protein accumulation by TNF-α combined with S100A12 in HASMCs was attenuated by sitagliptin. Western blotting assay showed the effects of the knockdown of TNFR-1 and RAGE proteins by these siRNAs. Compared with the TNF-α combined with S100A12-stimulated cells in the presence of scrambled siRNAs, any combination of TNFR-1 or RAGE siRNAs dramatically abolished TNF-α-stimulated calcification. *P < 0.05 compared with the control group, and ^#^*P* < 0.05 compared with the TNF-α combined with S100A12 groups. N = 6 for each set of experiments. **(c)** Sitagliptin attenuated the calcium deposition mediated by TNF-α combined with S100A12. Suppressed RAGE accumulation in HASMCs and siRNAs against RAGE-downregulated calcium deposition (*NAC* antioxidant agent, *SC* scramble, *TNF-R1* TNF-α receptor 1).

The DPP4 inhibitor had anti-atherosclerotic effects by reducing the production of ROS and inflammatory cytokines in adipocytes and aorta^33^. RAGE signaling was at least in part mediated by the oxidative stress from NADPH oxidase^21^. We used TNF-α + S100A12-treated HASMCs to investigate whether sitagliptin neutralized the oxidative stress and inhibited TNF-α-induced activation of NADPH oxidase. We incubated HASMCs with various doses of sitagliptin and found that sitagliptin did not inhibit the cell proliferation rate (Fig. 3a). The siRNA for RAGE significantly lowered the TNF-α + S100A12 induced expression of RAGE (Fig. 3b) and calcification (Fig. 3c) without affecting the expression of TNFR. The siRNA for TNFR-1 was very effective in blunting the TNF-α + S100A12 induced up-regulation of the RAGE and TNFR-1 expression (Fig. 3b). Sitagliptin treatment appeared to exert a dose-dependent effect on suppressing the TNF-α + S100A12-induced RAGE expression (Fig. 3b) and calcium deposits (Fig. 3c). Moreover, sitagliptin was as effective as NAC, in blunting the TNF-α + S100A12-induced calcium deposits (Fig. 3c).

### Sitagliptin blunts TNF-α + S100A12-induced HASMC oxidative stress, osteogenic marker expression, and calcification

Activated RAGE promoted the activation of NADPH oxidase^36^, which led to the generation of intracellular H_2_O_2_ in VSMCs^37^. To determine whether sitagliptin interrupted the RAGE signaling pathway by inhibiting NADPH oxidase activity, the measurements of intracellular H2O2 and NADPH oxidase activity were assayed.

**Figure 4:**
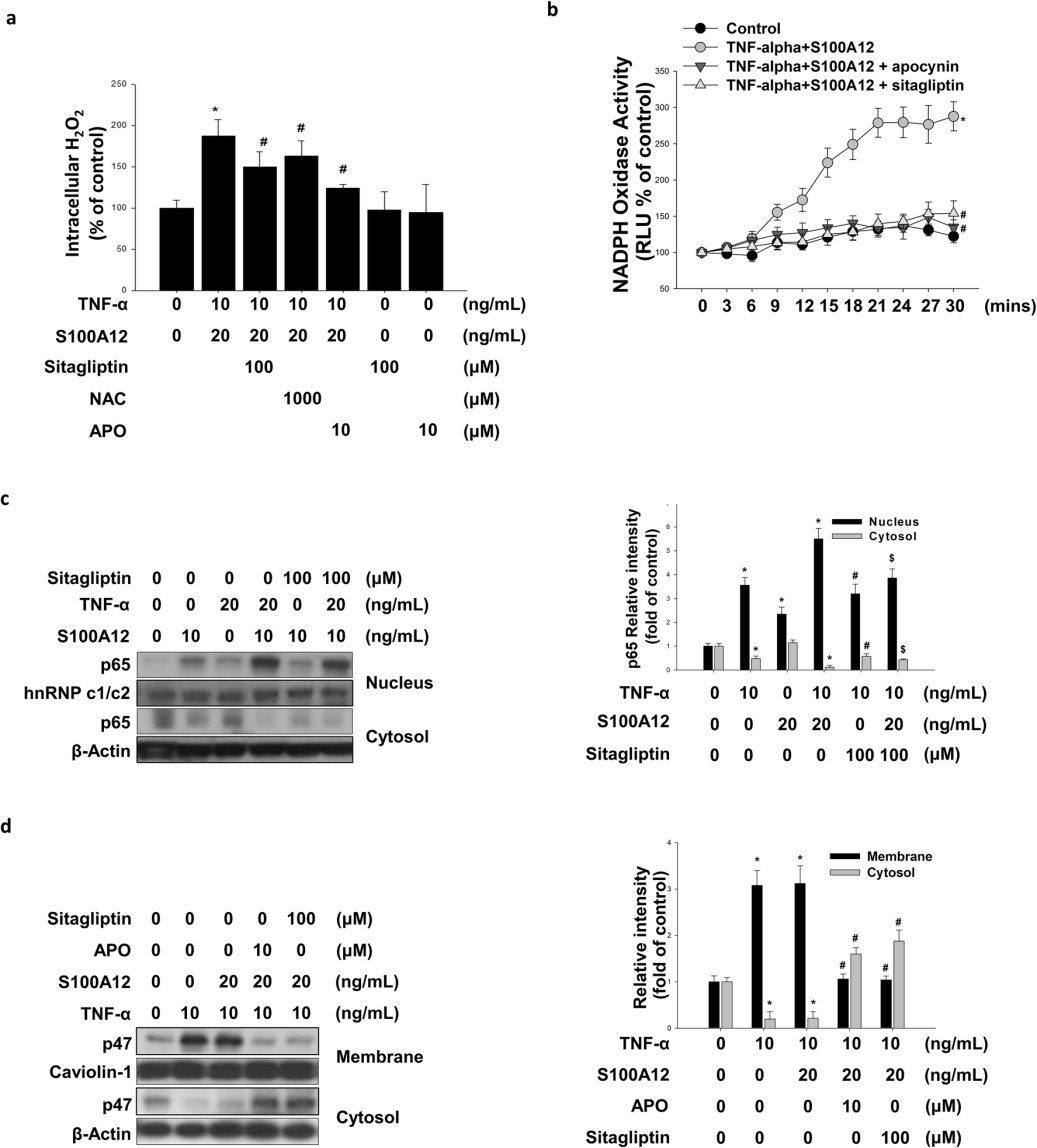
Sitagliptin attenuated TNF-α combination with S100A12-induced oxidative stress, NF-κB, and p47 activation in HASMCs. **(a)** HASMCs were cultured in an osteogenic differentiation medium for 1 day in the presence or absence of TNF-α combined with S100A12 concomitant with sitagliptin (50 µM), APO (apocynin: 500 µM; Nox inhibitor). Intracellular hydrogen peroxide generation was assessed by DCF-AM staining. **(b)** Nox activity was evaluated by lucigenin chemiluminescence. **(c)** The expression level of NF-κB subunit p65 (nucleus and cytosol fraction) was assessed by Western blotting. hnRNP c1/c2 was used as a nucleus fraction loading control. **(d)** The expression level of Nox subunit p47 (membrane fraction) was assessed by Western blotting. Caveoli-1 was used as a membrane fraction loading control. *P < 0.05 compared with the control group, and ^#^*P* < 0.05 compared with the TNF-α combined with S100A12 groups. N = 6 for each set of experiments.

We found that treatment with TNF-α + S100A12 stimulated H_2_O_2_ production and NADPH oxidase activity in HASMCs (Fig. 4a,b). Treatment with TNF-α alone or TNF-α + S100A12 promoted p65 migration from cytosol into nucleus (Fig. 4c) while stimulating p47 translocation from cell nucleus to cell membrane (Fig. 4d). In addition, treatment with sitagliptin (100 μM), NAC (1000 μM) or APO (10 μM) attenuated the TNF-α + S100A12-induced H_2_O_2_ generation and NADPH oxidase activation (Fig. 4a,b). Sitagliptin was also found to blunt the TNF-α + S100A12-induced migration of cytosolic p65 into the nucleus (Fig. 4c). Treatment of sitagliptin and APO were both effective in blunting the TNF-α + S100A12-induced migration of p47 translocating from cell nucleus to the cell membrane (Fig. 4d).

### Sitagliptin blunts TNF-α + S100A12-induced expression of osteogenic markers and calcification

**Figure 5:**
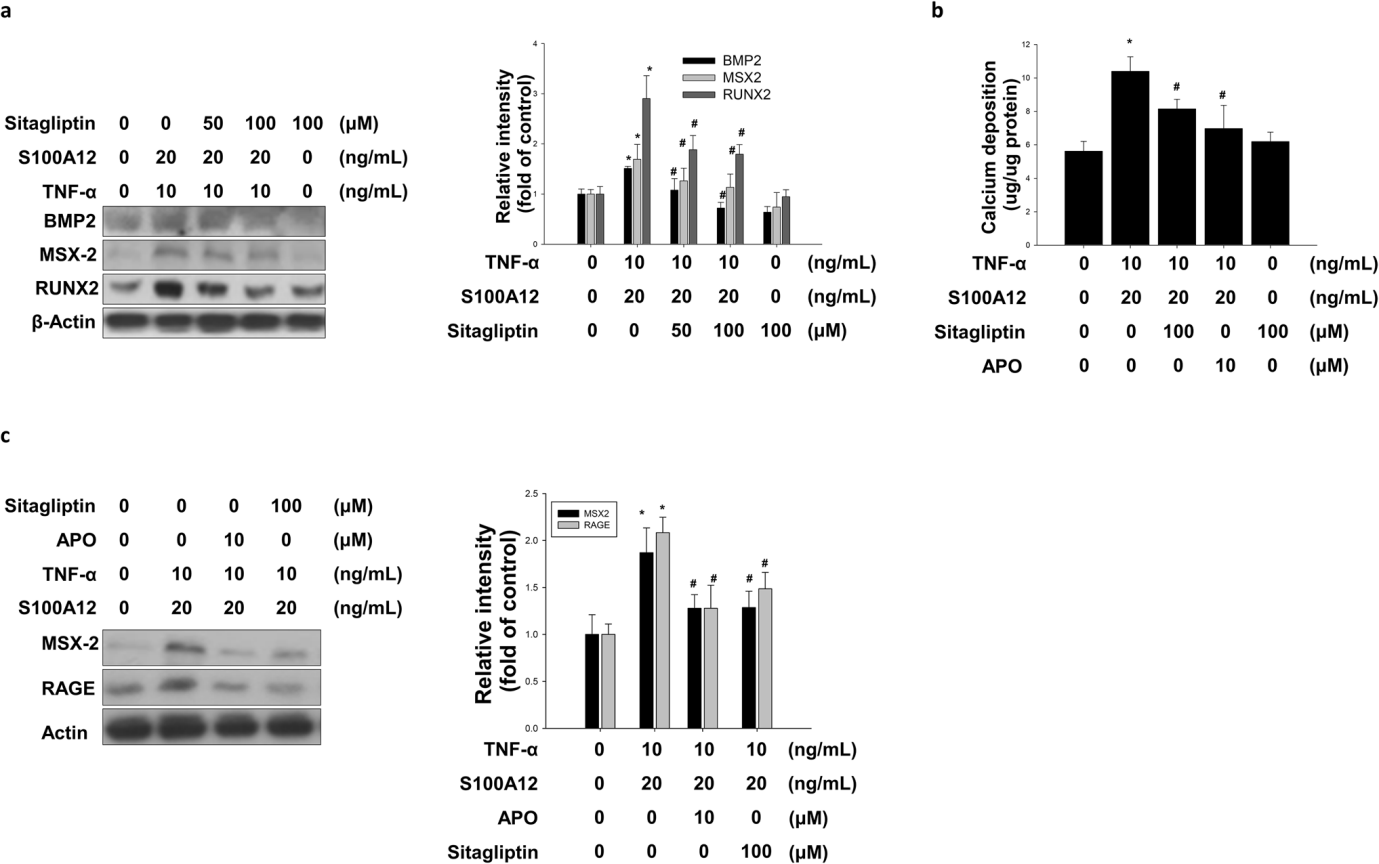
Sitagliptin modulated ROS generation and the expression of RAGE. **(a)** HASMCs were cultured in an osteogenic differentiation medium for 1 day in the presence or absence of TNF-α combined with S100A12 and concomitantly with sitagliptin. Sitagliptin blocked the induction of MSX2, BMP-2, and RUNX2 accumulation induced by TNF-α combined with S100A12. **(b)** Antioxidant agents (NAC and APO) attenuated the TNF-α combined with S100A12-induced bone marker MSX2 and **(c)** RAGE accumulation in HASMCs (sitagliptin; NAC: ROS scavenger; APO: Nox inhibitor). *P < 0.05 compared with the control group, and ^#^*P* < 0.05 compared with the TNF-α combined with S100A12 groups. N = 6 for each set of experiments.

To determine whether sitagliptin inhibited osteogenic marker expression and cell calcium deposition. We assayed that treatment with TNF-α + S100A12 increased the expression levels of BMP-2, MSX2 and RUNX2 and calcium deposit (Fig. 5a,b), whereas treatment with sitagliptin significantly blunted the TNF-α + S100A12-induced effects on HASMCs (Fig. 5a,b). Treatment with sitagliptin and APO were both effective in blunting the TNF-α + S100A12-induced expression of RAGE and MSX2 (Fig. 5c).

## Discussion

**Figure 6:**
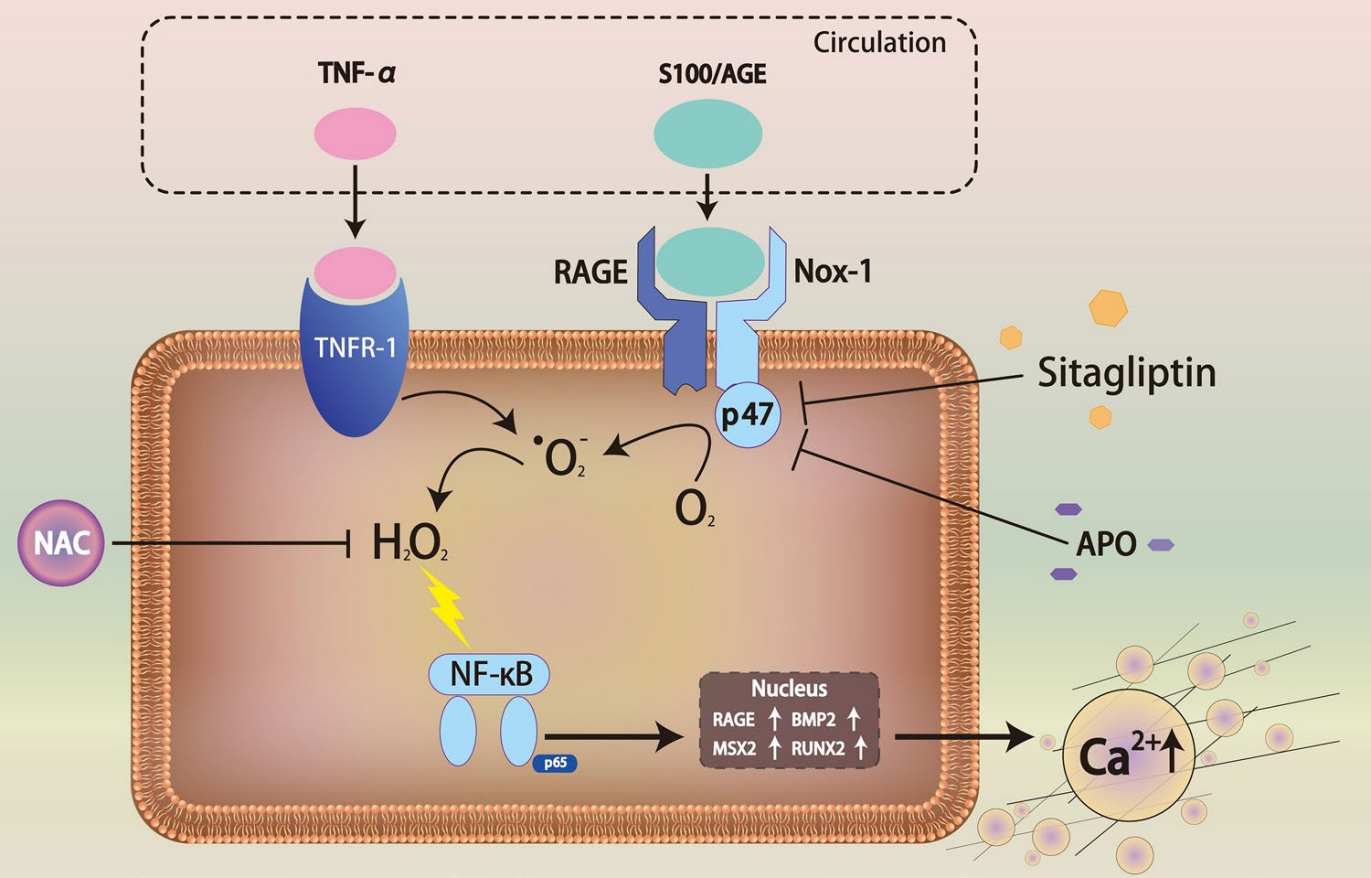
The proposed scheme of sitagliptin attenuates TNF-α + S100A12-induced VSMC calcification through inhibition of RAGE-Nox pathways. TNF-α enhances RAGE accumulation and activation by binding to the RAGE ligand, S100A12. S100A12 interacts with Nox1 NADPH oxidase to promote osteochondrogenic genes expression and calcium deposition in VSMCs via oxidative stress signaling. Sitagliptin reduces osteogenic mineralization by inhibiting the activation of NADPH oxidase and ROS production.

Hyperglycemia and dyslipidemia are the traditional risk factors of arterial calcification^38^. In this study, HFD-fed LDLR^−/−^ mice were used as a model system to investigate the potential effect of sitagliptin on T2DM patients’ initiation and progression of artery calcification. We found that HFD feeding caused obesity, hyperglycemia, hyperlipidemia and severe artery calcification on LDLR^−/−^ mice. HFD feeding also increased inflammation and oxidative stress as indicated by serum TNF-α and nitrotyrosin levels. Orally administration of sitagliptin (100 mg kg^−1^ day^−1^) moderately decreased the HFD-induced serum TNF-α (45% reduction) and nitrotyrosin (70% reduction), improved the levels of blood glucose and triglyceride, reduced the body weight, aortic calcium content and DPP4 activity, and significantly blunted the artery calcification. In-vitro studies showed that sitagliptin attenuated TNF-α and S100A12-induced calcium deposition and attenuated the expression of RAGE and osteogenic markers such as MSX2, BMP-2, and RUNX2. Sitagliptin reduced RAGE accumulation by downregulating the activated NF-κB translocation and attenuated ROS generation by reducing NADPH oxidase p47 subunit translocation. Data strongly suggest that the protective effect of sitagliptin may not be a consequence of its glucose lowering effect via the inhibition of DPP4. Instead, the protective effect of sitagliptin against calcification was provided by 1) blocking the activation of cell membrane surface NADPH, and 2) down-regulation of TNF-α and RAGE (Fig. 6).

Sitagliptin, a DPP4 inhibitor was the first commercialized approved for treatment of patients with T2DM in Oct 2006 in USA and in 2007 by the European Medicines Agency (EMA) at a dosage of 100 mg daily^39^. Sitagliptin is suggested to have a cardiovascular protective effect and glucose-lowering effect similar to saxagliptin and alogliptin^39^. Zheng et al. showed that treatment with 100 mg kg^−1^ day^−1^ of sitagliptin increased the mass and function of pancreatic β-cells, reduced the levels of fasting blood glucose, postprandial blood glucose and HbA1c in T2DM mice^40^. Previous studies reported that significance of sitagliptin on vascular protection in Zucker diabetic fatty rats. Sitagliptin decreased blood glucose concentrations and increased plasma insulin concentrations, augmented acetylcholine-induced vascular relaxation reduced the DPP4 activity, malondialdehyde levels, the expressions of p22^phox^ and monocyte chemoattractant protein-1^41^. Sitagliptin attenuated the progress of atherosclerosis in ApoE knockout mice via AMP-activated protein kinase (AMPK) and Mitogen-activated protein kinase (MAPK)-dependent mechanisms followed by reducing leukocyte-endothelial cell interaction and inflammation reactions. These vascular protection and anti-atherosclerotic effects of sitagliptin were mediated by attenuating oxidative stress and NF-κB signaling^32,42^.

The proinflammatory cytokine S100A12, a RAGE agonist, is associated with coronary atherosclerotic plaque rupture. S100A12 increased the atherosclerotic plaque size, expression of bone morphogenic protein and other osteoblastic genes in aorta and cultured vascular smooth muscle^23^, and promoted the VSMC osteochondrogenic mineralization in transgenic mice. This finding provided a strong evidence that the S100/RAGE axis enhanced vascular calcification^43^. The activities of S100A12 including chemotactic activity and activation of intracellular signaling cascades led to induction of cytokine production and oxidative stress^44^. In addition, Nox inhibition reduced osteogenic programming and calcification^23^. The signaling of S100A12 was controlled by the presence of RAGE and oxidative stress signaling. In this study, HFD-induced the mice hyperglycemia and dyslipidemia contributed to arterial calcification, whereas these were significantly downregulated after 24 weeks-treated sitagliptin (Fig. 1b, Table 1). A previous study showed that sitagliptin attenuated body weights and reduced local inflammation in adipose tissue of HFD-induced obese mice^45^. We suggested that sitagliptin decreased body weight gain by attenuating the levels of TNF-α and TG (Fig. 1a, Table 1). Moreover, DPP4 induced valvular calcification and promoted calcific aortic valve disease progression by inhibiting autocrine insulin-like growth factor-1 (IGF-1) signaling. Treatment with sitagliptin reduced the calcium deposits and increased plasma IGF-1 levels^46^. In our study, sitagliptin significantly reduced HFD-induced DPP4 activity consequently attenuated arterial calcification in LDLR^−/−^ mice (Fig. 1c,d). GLP-1 was known to be involved in anti-inflammation^47^. Sitagliptin dietary supplementation reduced adiposity and improved glucose metabolism in obese and diabetic mice, which was associated with GLP-1 elevation^48^. Sitagliptin boosted the circulatory GLP-1 levels by retarding the degradation of GLP-1^49^. However, GLP-1 did not reduced the TNF-α-induced calcification in HASMCs (Fig. S2). We suggested that GLP-1 played a minor role in blunting of arterial calcification on HFD-fed LDLR^−/−^. Furthermore, sitagliptin attenuated the accumulation of RAGE and arterial calcification on HFD-induced LDLR^−/−^ mice (Fig. 1e). These results suggested that sitagliptin attenuated HFD-induced arterial calcification by improvement lipid profile, anti-inflammation, and reducing the expression of RAGE on LDLR^−/−^ mice.

We next employed HASMCs to clarify the underlying mechanisms of HFD-induced arterial calcification. It has been known that TNF-α plays crucial role in HFD-induced arterial calcification^13^. HFD increased the accumulation of circulatory S100A12 in vivo. In addition, stress-mediated VSMC expressed S100A12 and overexpression of S100A12 accelerated arterial calcification in ApoE^−/−^ mice^23^. The accumulation of S100A12 could be induced by TNF-α in HASMCs, whereas there was no effects with recombinant S100A12 treatment alone (Fig. 2a,b). Previously study suggested S100/calgranulins were endogenously expressed in granulocytes and myeloid cells, and were induced in VSMCs of the atherosclerotic vessel^50^. S100A12 augmented the atherosclerosis-triggered osteogenesis and TNF-α increased the expression of RAGE. Similar to the previous studies, treatment of TNF-α or S100A12 significantly increased the accumulation of RAGE and calcium deposition in HASMCs (Fig. 2c–e). RAGE activated the proinflammatory transcription factor NF-κB. Interestingly, activated NF-κB caused sustained RAGE expression and created a positive-feedback loop^21^. ApoE^−/−^/S100A12 transgenic mice-isolated VSMCs treated with the condition medium (contain macrophages and serum isolated from hyperlipidemic ApoE^−/−^ mice) increased oxidative stress, the expression of osteogenic marker and calcification^23^. These findings suggest that the ROS or inflammatory cytokines from the macrophages in hyperlipidemic ApoE^−/−^ mice might activate a mechanism which synergistically interacts with the RAGE/S100A12 signaling in stimulation of arterial atherosclerosis and calcification. We employed a combined strategy to mimic the in-vivo condition. There was no cell toxicity at the dosage of 100 μM (Fig. 3a). Treatment with TNF-α + S100A12 significantly induced the expression of RAGE and calcium deposition (Fig. 3b,c). Sitagliptin was found to exert a dose response on depressing the stimulation of TNF-α + S100A12. These results suggested that combination of TNF-α with S100A12 had synergetic effects to trigger calcium deposition in HASMCs.

Inflammation increased the calcium deposition through NADPH oxidase-activated regulated ROS productions^34^. ROS products were generated by mitochondrial oxidases, Nox, and nitric oxide synthases^16^. The NADPH oxidase is composed of Nox, p22^phox^ and p47^phox^. It has been reported that activation of RAGE induced the expression of Nox-1, Nox-4, p22^phox^ and p47^phox^ and increase ROS production in VSMCs^51^. Generally, superoxide generated by NADPH oxidase was short-lived and was rapidly transferred to H_2_O_2_ by superoxide dismutase^52^. To investigate whether sitagliptin attenuated ROS productions such as H_2_O_2_ with the combination of TNF-α and S100A12. We measured the TNF-α + S100A12-induced H_2_O_2_ production and NADPH oxidase activity after NAC, APO and sitagliptin treatment. As a result, combination of TNF-α and S100A12 increased ROS generation, which were significantly decreased by sitagliptin (Fig. 4a,b). We suspected that sitagliptin contributed its protective against HASMCs calcification by inhibiting NADPH oxidase activity.

Translocation of p47 from HASMCs cytosol to membrane indicates activation of NADPH oxidase and the migration of p65 from cytosol into nucleus indicates activation of NF-κB. It is known that suppression of the membrane translocation of p47phox was shown to inhibit the assembly of NADPH oxidase by alpha tocopherol^53^. We further investigated the p47 and p65 migration in HASMCs with TNF-α + S100A12, TNF-α + S100A12 + sitagliptin and TNF-α + S100A12 + APO treatment. Combination of TNF-α and S100A12 increased p47 translocation to the cell membrane, whereas p47 translocation was decreased by sitagliptin treatment (Fig. 4c,d). Previous studies have suggested RUNX2 plays a crucial role in oxidative stress-induced VSMC calcification and SMC-specific RUNX2 deficiency inhibited vascular calcification^54,55^. Another study demonstrated that sitagliptin attenuated oxidative stress in diabetic rats^56^. We found that sitagliptin attenuated TNF-α + S100A12-induced calcium deposition and the expression of osteogenic markers protein accumulation via anti-oxidation (Fig. 5a–c).

There were several study limitations. First, we demonstrated that inhibition of RAGE decreased the expression of osteogenic markers and subsequently inhibited HASMCs calcification. We also found that inhibition of the TNF-R1 decreased the expression of RAGE. These findings suggested that TNF-R1 might be the upstream regulator of RAGE in TNF-α/NF-κB pathway. The mechanism of TNF-R1 silence-suppressed accumulation of S100A12-induced RAGE and the relationship between sitagliptin and TNF-R1 were still unknown. Second, this study could not rule out the protection effect of sitagliptin by elevating GLP-1. It has been known DPP4 inhibitors improved glucose metabolism in diabetic patient, which was associated with GLP-1 elevation. Third, we demonstrated that sitagliptin attenuated HFD-induced calcification in LDLR^−/−^ mice. The effects of sitagliptin treatment on HFD-supplemented wild-type mice should be further investigated.

In summary, in addition to the known roles of DPP4 inhibitors in influencing glucose levels and other pleiotropic effects, such as anti-inflammatory and anti-oxidative effects, we demonstrated that sitagliptin acts via a novel mechanism, independent of its blood glucose-regulating effect, to prevent arterial calcification.

## Conclusion

In addition to the known roles of DPP4 inhibitors in influencing glucose levels and other pleiotropic effects, such as anti-inflammatory and anti-oxidative effects, we demonstrated that sitagliptin acts via a novel mechanism, independent of its blood glucose-regulating effect, to prevent arterial calcification.

## Materials and methods

### Materials

Human SMC growth medium (M231), smooth muscle growth supplement, trypsin/ethylenediaminetetraacetic acid (EDTA) solution, trypsin neutralizer solution, and HASMCs were obtained from Cascade Biologics (Portland, OR). Fetal bovine serum (FBS), antibiotic–antimycotic mixture, mouse TNF-α kits, oligofectamine, and 2’, 7’-dichlorofluorescein diacetate were obtained from Life Technology (Grand Island, NY). Small interfering RNA (siRNA) oligonucleotides against RAGE and antibodies against human TNFR-1 (sc-8436), MSX2 (sc-17729), BMP-2 (sc-6895), RUNX2 (sc-10758), CD68 (sc-9139), β-actin (sc-47778), and anti-hnRNP c1/c2 (sc-32308) were obtained from Santa Cruz Biotechnology (Santa Cruz, CA). Antibodies against human Nox subunit p47 (#610354), and caveoli-1 (#610406) were obtained from BD Biosciences (San Jose, CA). Unless otherwise specified, all other chemicals and reagents obtained from Sigma-Aldrich (St. Louis, MO). All methods in this study were reported in accordance with ARRIVE guidelines.

### Animal study

The eight-week-aged male LDLR^−/−^ mice (Jackson Labs #002207; C57BL/6 J background) were randomized into 3 groups: (1) normal diet group (Picolab Rodent Diet 20 #5053: 5% fat, 21% protein, 3.3% sucrose, and 28% starch; N = 6), (2) HFD group (Harlan Teklad, Diet TD88137 (21% milk fat (42% fat calories), 34% sucrose, and 0.15% cholesterol); N = 6) and (3) HFD + sitaglptin group (N = 6). Mice in group 1 and 2 were daily gavaged with 100 μL of distilled water. Mice in group 3 were given 100 mg kg^−1^ day^−1^ of sitagliptin by gavaging with 100 μL of sitagliptin solution (BioVision Research Products, Milpitas, CA). The timeline is shown in Fig. S1. Mice were fed a normal diet or high-fat diet and given water or sitagliptin for 24 weeks. All mice were kept in microisolator cages under a 12 h day/night cycle. The entire animal was given free access to chow and water. No inclusion and exclusion criteria were set and cofounders were not controlled. For each animal, three different investigators were involved as follows: the first investigator (CP) randomized the mice. This investigator was the only person aware of the treatment group allocation. A second investigator (PH) was responsible for the feeding. Finally, a third investigator (CY and JS) collected samples. The Institutional Animal Care and Use Committee of Taipei Veterans General Hospital approved the experiments (IACUC no.2020-265), including any relevant details. All animal study experiments were performed in accordance with the Guide for the Institutional Animal Care and Use Committee of Taipei Veterans General Hospital and the Guide for the Care and Use of Laboratory Animals of the US National Institutes of Health (8th edition, 2011). The LDLR^−/−^ mice were sacrificed after 24 weeks of treatments with exsanguination under anesthesia (100 mg^−1^ kg^−1^ ketamine–HCl and 20 mg^−1^ kg^−1^ xylazine via IP injection) after 6 h of fasting. The animals were considered adequately anesthetized when no attempt to withdraw the limb after pressure could be observed. The thoracic cavity was opened for blood and aorta (from heart to diaphragm) sample collections.

### Histology and immunohistochemistry

Aorta samples were cut into 4 sections and processed for histological staining, as described in our previous study^34^. Paraffin sections (5 µm) from the dissenting aorta were stained using various agents for semi-quantification of atherosclerotic lesion size and severity (hematoxylin and eosin (H&E) staining) and aortic calcium deposition (alizarin red S staining). Immunohistochemical (IHC) staining of RAGE and VSMC actin (SM α-actin) was performed as previously described^57^.

### Cell culture and cell viability assay

HASMCs were purchased from Life Technology (Grand Island, NY; Catalog number C0075C). The cells were grown and passaged as described previously^34^. Briefly, the HASMCs were grown in M231 medium containing SMC growth supplements and a 1% antibiotic–antimycotic mixture in an atmosphere of 95% air and 5% CO_2_ at 37 °C in plastic flasks. At confluence, the cells were subcultured at a ratio of 1:3, and passages 3 through 8 were used. The cytotoxicity of S100A12 protein and sitagliptin on HASMC cell viability were measured with the 3-(4,5-dimethylthiazol-2-yl)-2, 5-diphenyl tetrazolium bromide (MTT) assay.

### Soluble DPP-4 activity measurement

Serum DPP-4 activity was measured using the Enzyme-linked immunosorbent assay (ELISA) kit from Abcam (Abcam, Cambridge, MA, USA, ab22287) according to the manufacturer’s instructions.

### Quantification of aorta or cultured HASMC calcium deposits

Cultured cell Calcium content was determined using a BioChain Calcium Kit (BioChain, Hayward, CA, USA) as previously described^58^. Briefly, the 6 well plate cultured cells were counted and demineralized with 250 µL 0.6 N HCl for 12 h. A working reagent was prepared by mixing 75 µL reagent A and 75 µL reagent B and was equilibrated to room temperature before use. A volume of 50 µL diluted standards or samples were transferred to each well of a clear-bottom 96-well plate. Then, 200 µL working reagent was added, and the solution was mixed by light tapping. After incubation for 3 min at room temperature, absorbance was measured at 570–650 nm with a 96-well reader. The units of these results are ug mL^−1^ calcium. The other 6 well plate cultured cells were counted and solubilized in a 200 µL lysis solution containing 0.1 N NaOH and 0.1% sodium dodecyl sulfate (SDS) at room temperature for 5 min. Protein concentration was measured with a Bio-Rad DC Protein Assay Kit. The calcium content of the cultured cells normalized to the protein content was reported.

The aortic segments from experimental mice were extracted using 0.6 N HCl for 24 h and the calcium content of the extracts was determined using a BioChain Calcium Kit (BioChain, Hayward, CA, USA) as previously described^34^. The results were expressed as µg mg^−1^ of wet aortic tissues.

### Extraction of cellular proteins

Nuclear protein extracts were prepared, as previously described^59^. In brief, after cells were washed twice using ice-cold PBS, they were scraped off the plates with a cell scraper and dispersed in 1 mL ice-cold buffer A (10 mM HEPES/NaOH, pH 7.9, 10 mM KCl, 1.5 mM MgCl_2_, 1 mM dithiothreitol (DTT), 0.5 mM phenylmethylsulfonyl fluoride (PMSF), 2 µg mL^−1^ aprotinin, 2 µg mL^−1^ pepstatin, and 2 µg mL^−1^ leupeptin). Cells were harvested after centrifugation at 500×*g* for 10 min at 4 °C then cells were re-suspended in 80 µL buffer B (buffer A containing 0.1% Triton X-100) by gentle pipetting. The cell lysates were allowed to stand on ice for 10 min and then centrifuged at 12,000×*g* for 10 min at 4 °C. Nuclear pellets were re-suspended in 70 µL ice-cold buffer C (20 mM HEPES/NaOH, pH 7.9; 1.5 mM MgCl_2_; 1 mM DTT; 0.2 mM EDTA; 420 mM NaCl; 25% glycerol; 0.5 mM PMSF; 2 µg mL^−1^ aprotinin; 2 µg mL^−1^ pepstatin; 2 µg mL^−1^ leupeptin), incubated on ice for 30 min with intermittent mixing, and then centrifuged at 15,000×*g* for 30 min at 4 °C.

Cell membrane fractions were prepared, as described previously^34^ with some modifications. Briefly, HASMCs were lysed in a lysis buffer (10 mM Tris–HCl, 1 mM EDTA, 1 mM PMSF, 10 µg mL^−1^ aprotinin, and 0.5 µg mL^−1^ leupeptin; pH 7.5). The cell lysates were centrifuged at 3000×*g* for 20 min. Pellets were re-suspended in a lysis buffer (20 mM Tris–HCl, 150 mM NaCl, 1 mM EDTA, 1 mM ethylene glycol tetraacetic acid, 1% Triton, 2.5 mM sodium pyrophosphate, 1 mM β-glycerophosphate, 1 mM Na_3_VO_4_, 1 µg mL^−1^ leupeptin, and 1 mM PMSF; pH 7.5) and designated as the membrane fraction.

Total cell lysates were prepared in a lysis buffer (20 mM Tris–HCl, 150 mM NaCl, 1 mM EDTA, 1 mM ethylene glycol tetraacetic acid, 1% Triton, 2.5 mM sodium pyrophosphate, 1 mM β-glycerophosphate, 1 mM Na_3_VO_4_, 1 µg mL^−1^ leupeptin, and 1 mM PMSF; pH 7.5). The protein concentrations were determined with the Bio-Rad Protein Assay Reagent (Bio-Rad, Hercules, CA), and the samples were stored at − 70 °C.

### Western blot analysis

Western blot analysis was used to quantify the HASMCs nuclear and cytosol of the Nox p47phox, NF-κB p65, nuclear MSX2, BMP-2 and RUNX2. Proteins of interest were isolated by SDS polyacrylamide gel and transferred to polyvinylidene fluoride membranes (PVDF, Millipore, Bedford, MA). The PVDF was blocked with 5% milk solution (Skimmed instant milk powder with PBS-T) then probed with anti-p47phox, anti-TNFR-1, anti-p65, goat anti-MSX2, BMP-2, or RUNX2 (1:1000) antibodies. Then, they were incubated with horseradish peroxidase-conjugated secondary antibodies. The proteins were visualized using an enhanced chemiluminescence detection kit (Amersham Biosciences, Piscataway, NJ). Anti-β-actin (1:5000), anti-caveolin-1 (1:1000), and anti-hnRNP c1/c2 (1:1000) antibodies were used as loading controls. All western blot images were detected using the Amersham Imager 680 (Cytiva, Marlborough, MA, USA). Protein expression levels were quantified as optical densities using ImageJ software (NIH Image software).

### Nox activity assay and hydrogen peroxide determination

Nox activity was determined with superoxide-dependent lucigenin chemiluminescence, as previously described^34^. Confluent HASMCs in 6-well plates were pretreated with various concentrations of antioxidant reagents followed by treatment with TNF-α + S100A12 with or without sitagliptin for 1 day. Cell membrane extract (40 µg) and 5 µM dark-adapted lucigenin were added to a 96-well luminometer plate and adjusted to a final volume of 250 µL with oxidase assay buffer before 100 µM NADPH was added. Relative light units (RLUs) were measured with a luminometer (Dynatech ML2250, Dynatech Laboratories Inc., VA). Light emission was recorded every 3 min for a total of 30 min and expressed as mean RLUs min^−1^.

The ROS production in HASMCs was determined by fluorometric assay using dichloro-dihydro-fluorescein diacetate (DCFH-DA) as the probe. This method was based on the oxidation by H_2_O_2_ of nonfluorescent DCFH-DA to fluorescent 2’, 7’-dichlorofluorescin. Confluent HASMCs in 24-well plates were pretreated with various concentrations of antioxidant reagents followed by treatment with TNF-α + S100A12 with or without sitagliptin for 1 day. The cells were washed with PBS, and then 250 μL of serum-free M231 containing 10 µM DCFH-DA was added to the well for 30 min. The fluorescence intensity (relative fluorescence units) was measured at 485 nm excitation and 530 nm emission using a fluorescence microplate reader after the plates were incubated for 45 min at 37 °C.

### siRNA transfection

siRNA oligonucleotides against RAGE were suspended in RNase-free water at a concentration of 10 µM. Cells were seeded one day before transfection to ensure HASMCs were 85%–95% confluent on the day of transfection. For transfection, the regular cell culture medium was replaced with a serum-free medium without antibiotics. The cells were transfected with siRNA using oligofectamine at a ratio of 1 siRNA: 2 oligofectamine (µg:µL) at a final concentration of 25–50 nM siRNA. The cells were incubated with the siRNA–oligofectamine complex for 5 h. Then, the serum-free medium was replaced with a normal medium (containing 10% FBS) without antibiotics, and the cells were incubated for 48 h before further analysis.

### Statistical analyses

Data were expressed as mean ± standard deviation (SD). Statistical evaluation was performed using Student’s *t*-test or one-way analysis of variance, followed by Dunnett’s test. A *P* value of < 0.05 was considered significant.

## Supplementary Information

Supplementary Information.

## Data Availability

The datasets used and/or analyzed during the current study are available from the corresponding author on reasonable request.

## Abbreviations

AGEs: Advanced glycation end products
APO: Apocynin
ApoE: Apolipoprotein-E
BMP-2: Bone matrix protein-2
T2DM: Type 2 diabetes mellitus
DPP4: Dipeptidyl peptidase-4
LDLR: Low-density lipoprotein receptor
MSX2: Msh homeobox 2
NADPH: Nicotinamide adenine dinucleotide phosphate
Nox: Nicotinamide adenine dinucleotide phosphate oxidase
NF-κB: Nuclear factor (NF)-κB
ROS: Reactive oxygen species
RAGE: Receptor for advanced glycation end products
RUNX2: Runt-related transcription factor 2
VSMCs: Vascular smooth muscle cells
TNF: Tumor necrosis factor
GLP-1: Type I glucagon-like peptide
