# Supplementary material for: Sitagliptin attenuates arterial calcification by downregulating oxidative stress-induced receptor for advanced glycation end products in LDLR knockout mice
Source: Sci Rep. 2021 Sep 8;11:17851. doi: 10.1038/s41598-021-97361-w (PMC8426400; doi:10.1038/s41598-021-97361-w)
Supplement: Supplementary file 1 — Supplementary Information. [file 41598_2021_97361_MOESM1_ESM.pdf]

Supplementary Figures

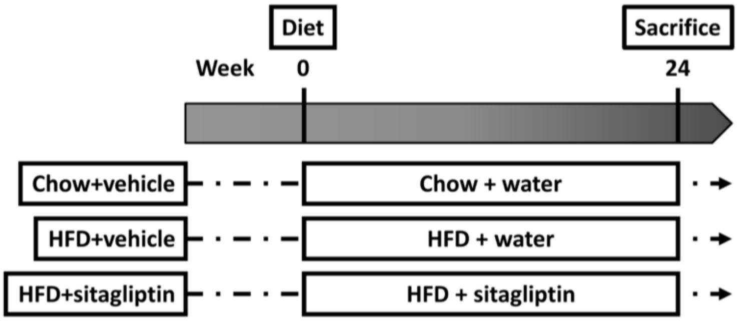

S-Fig. 1. Study timelines.

The eight-week-aged male LDLR$^{-/-}$ mice were randomized into 3 groups: 1) normal diet group (N=6), 2) HFD group (N=6) and 3) HFD+sitaglptin group (N=6). Mice in group 1 and 2 were given daily with 100 μL of distilled water. Mice in group 3 were given 100 μL of 100 mg^{-1}Kg^{-1}day^{-1} sitagliptin solution.

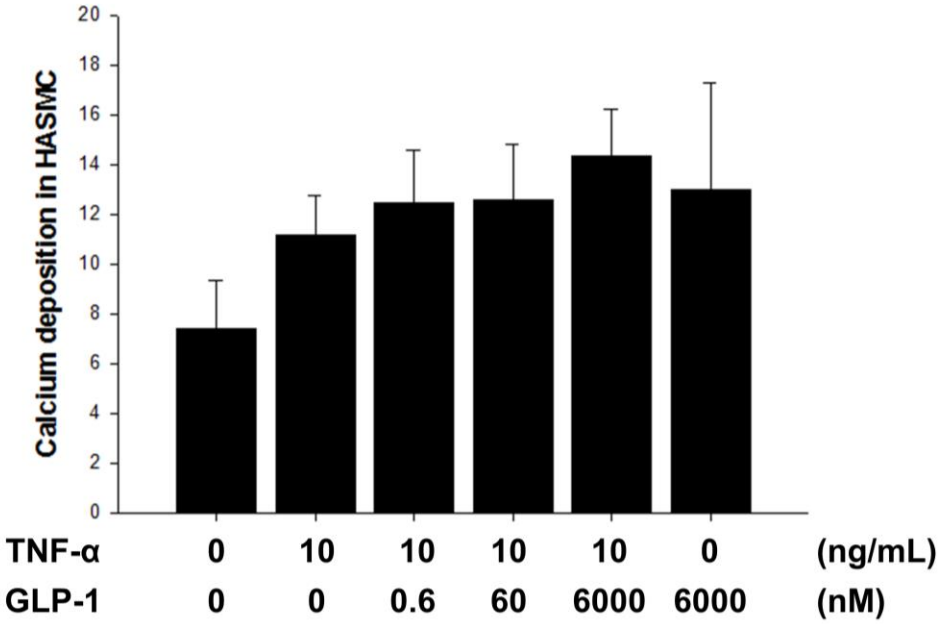

S-Fig. 2. Effect of GLP-1 attenuated TNF-α-induced calcium deposition in HASMCs.

HASMCs were cultured in osteogenic differentiation medium treatment with TNF-α for 4 days in the presence or absence of GLP-1 for 1 day. Calcium deposition was induced dose dependently by TNF-α for 4 days.

Fig. 2a

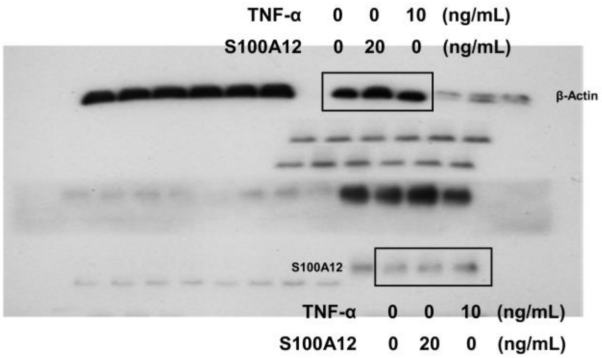

Fig. 2c

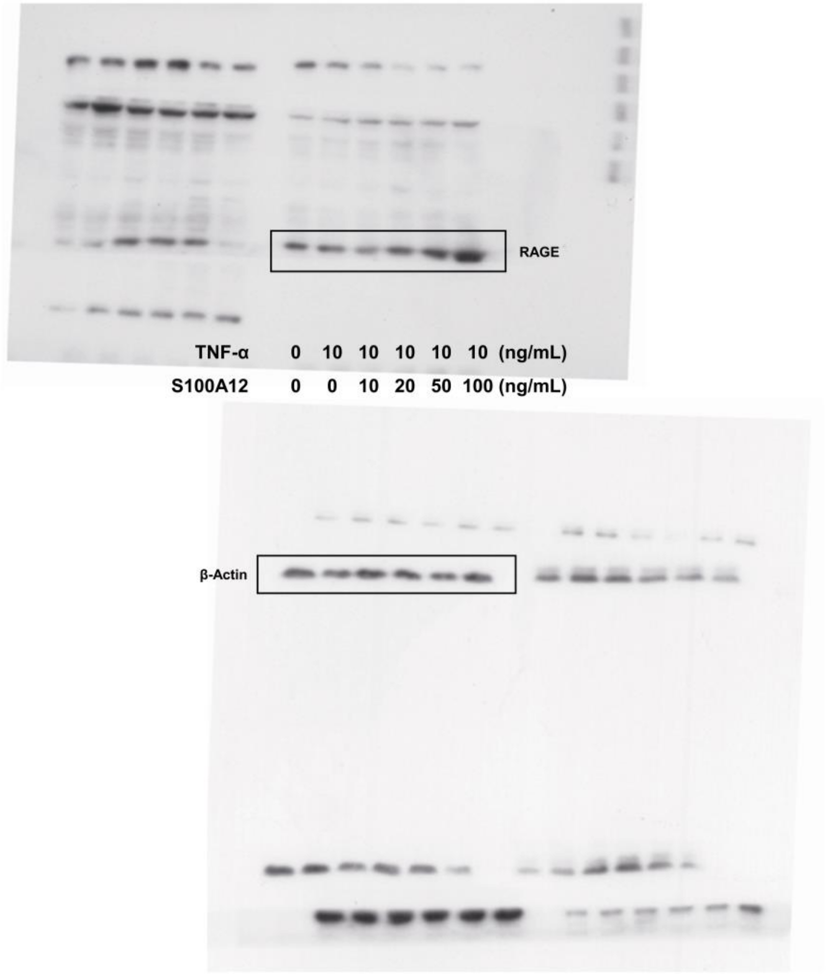

S-Fig. 3. Original full-length gel images of S100A12, RAGE and β-actin in Figure 2a and 2c.

Fig. 2d

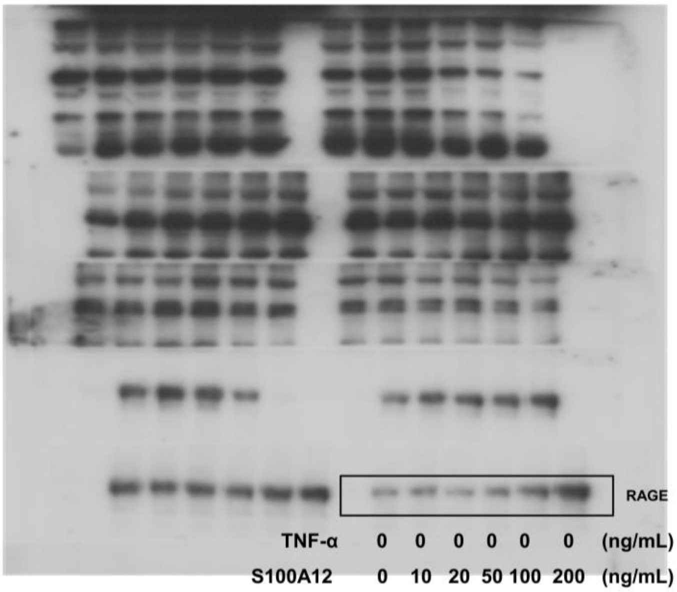

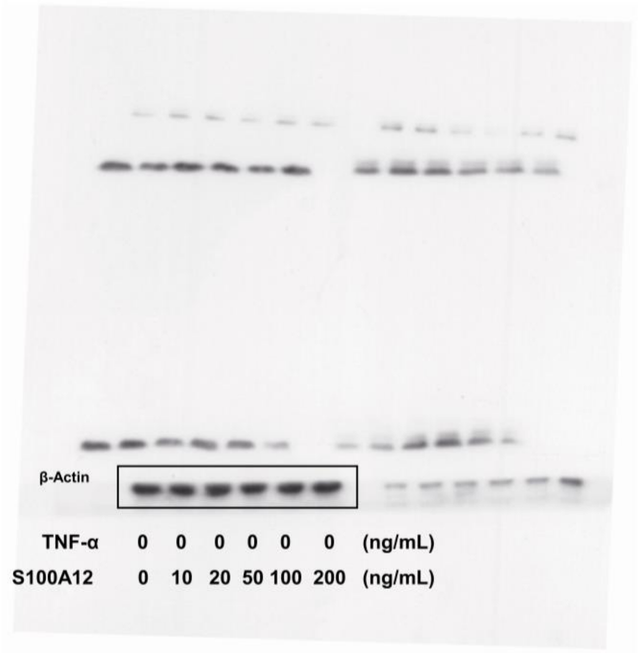

S-Fig. 4. Original full-length gel image of RAGE and β-actin in Figure 2d

Fig. 3b

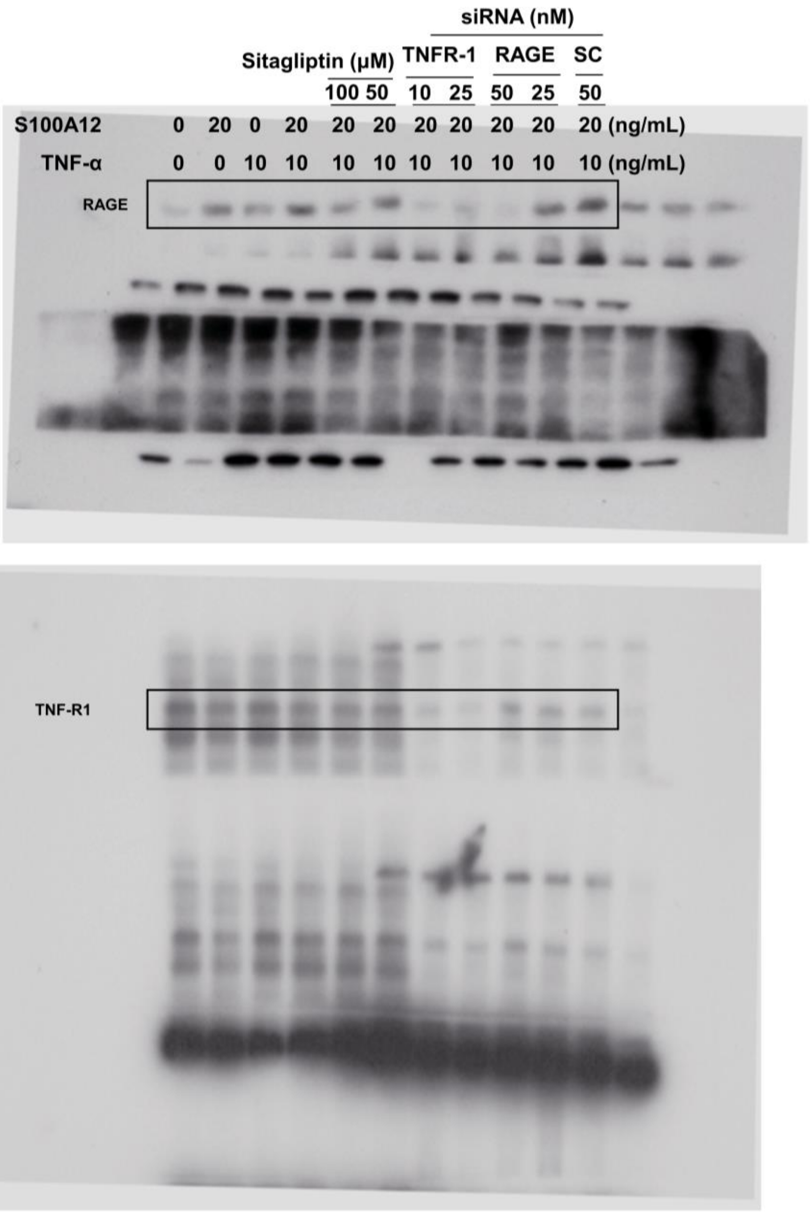

S-Fig. 5. Original full-length gel images of RAGE and TNF-R1 in Figure 3b.

Fig. 3b

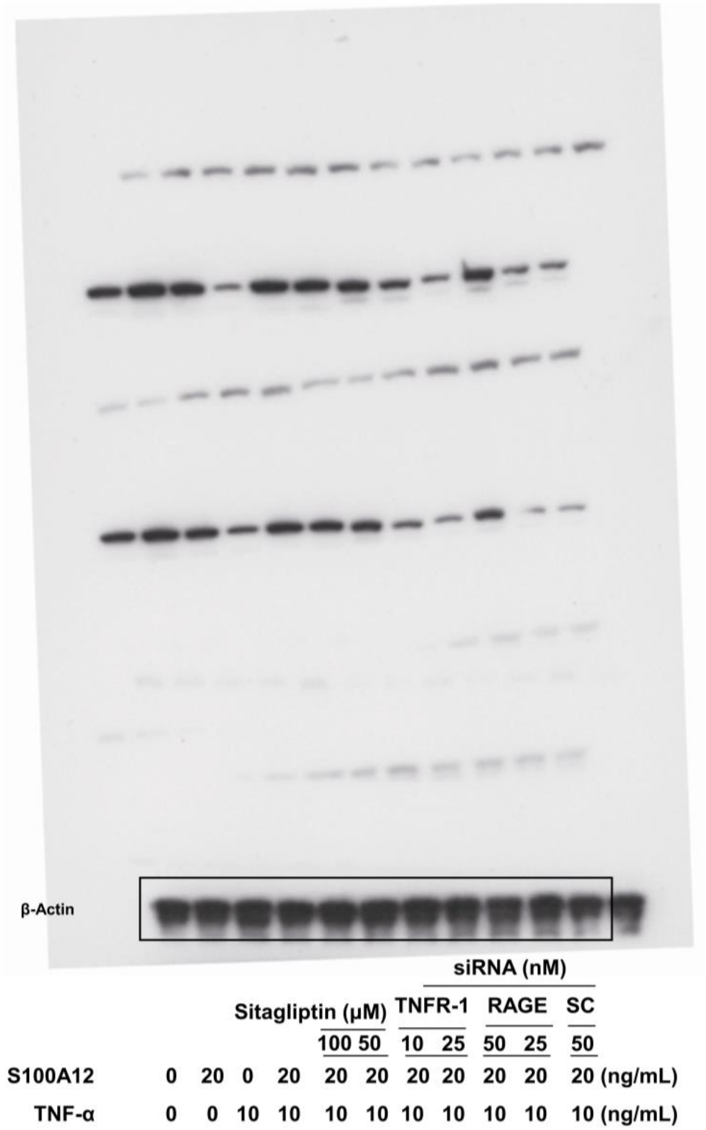

S-Fig. 6. Original full-length gel image of β-actin in Figure 3b.

Fig. 4c

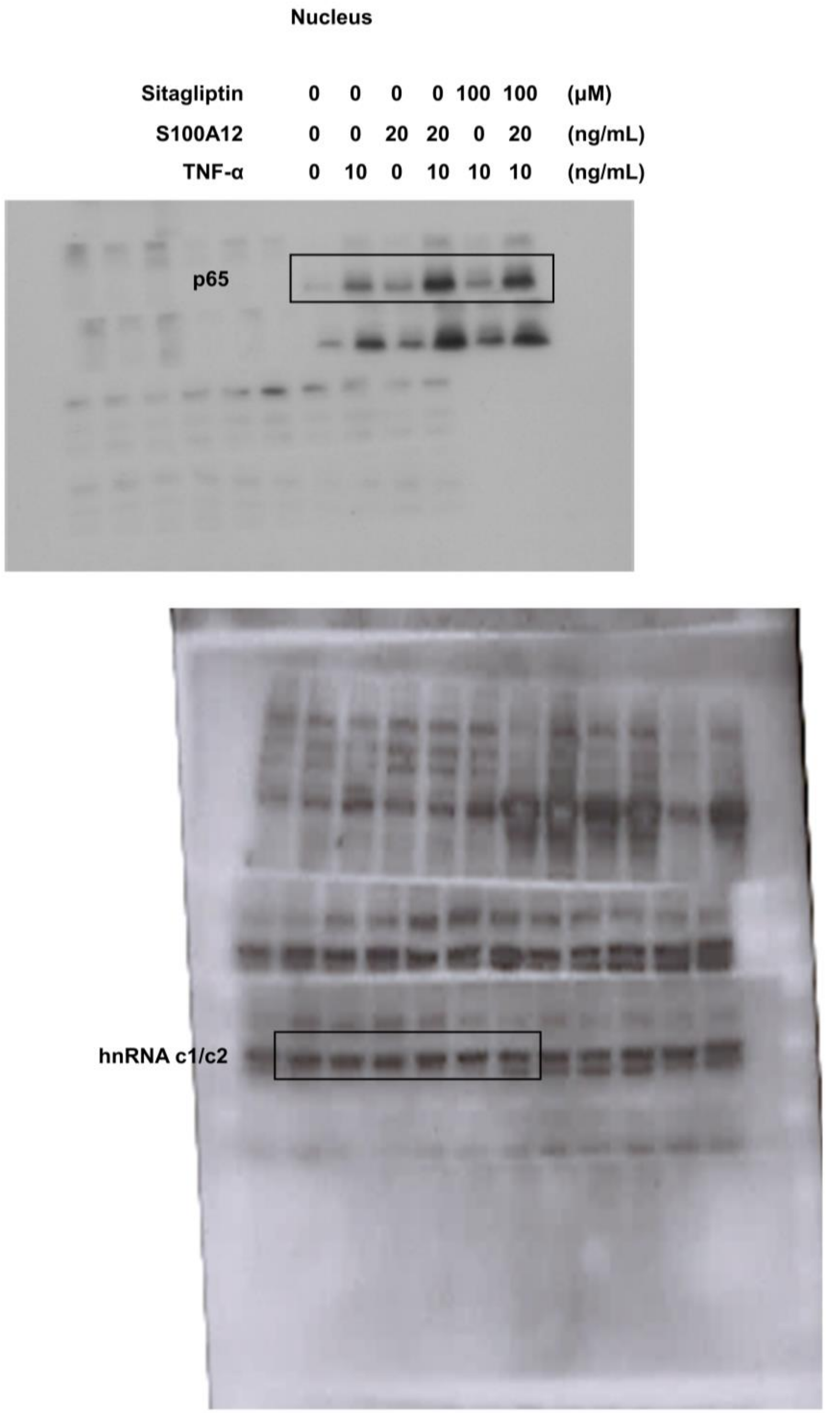

S-Fig. 7. Original full-length gel images of p65 and hmRNA c1/c2 in Figure 4c.

Fig. 4c

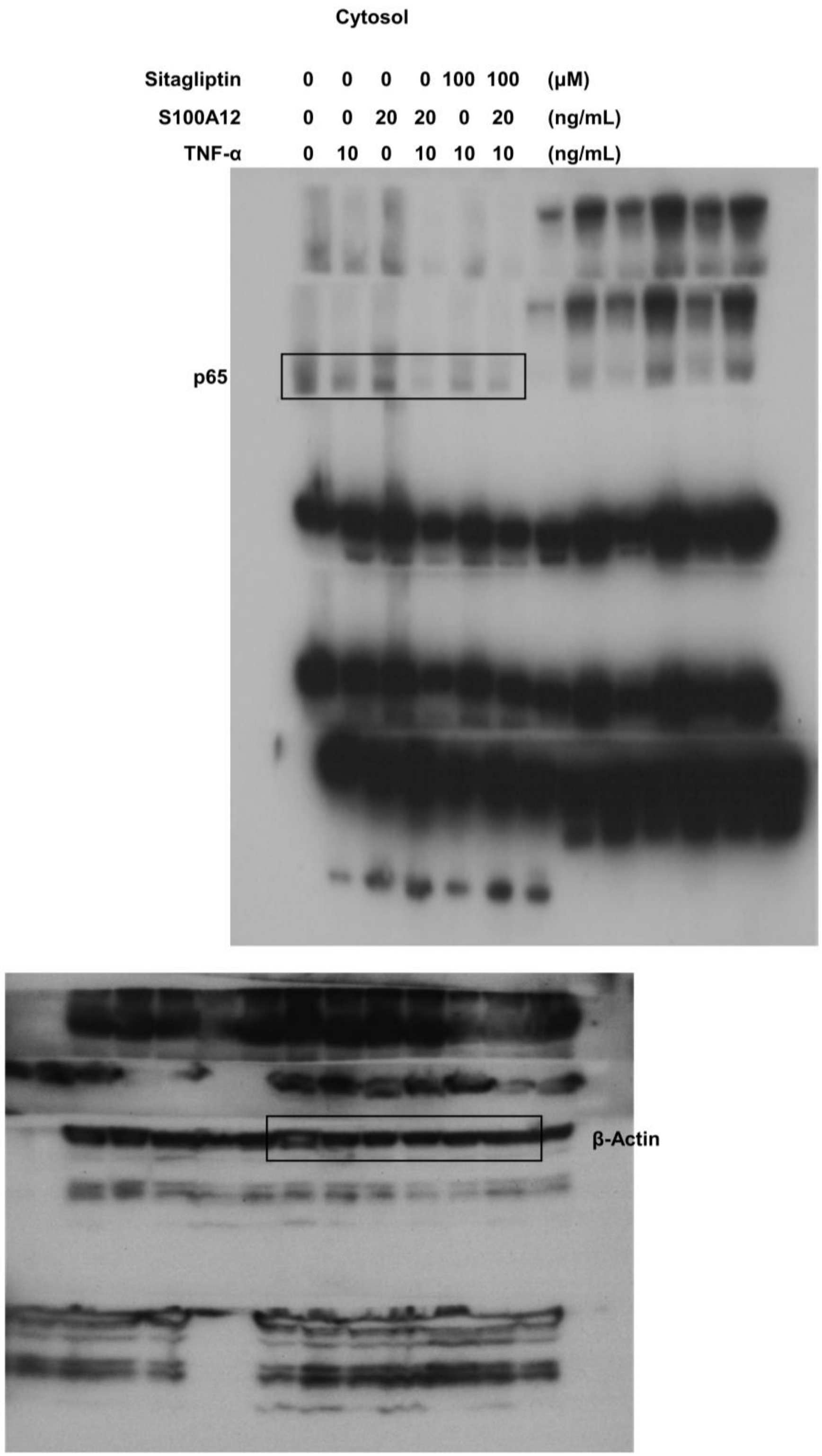

S-Fig. 8. The Original full-length gel images of p65 and β-actin in Figure 4c.

Fig. 4d

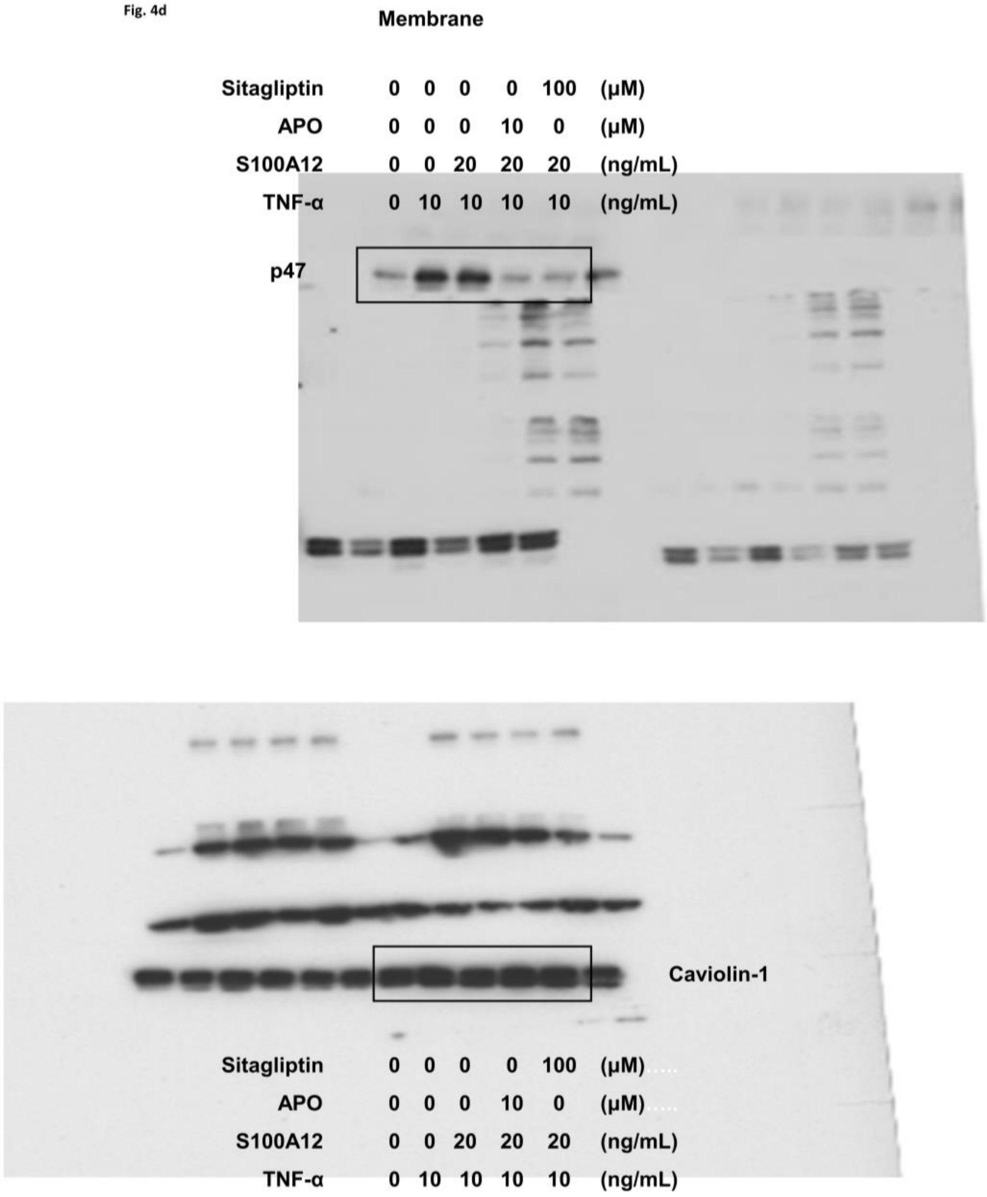

S-Fig. 9. Original full-length gel images of p47 and caviolin-1 in Figure 4d.

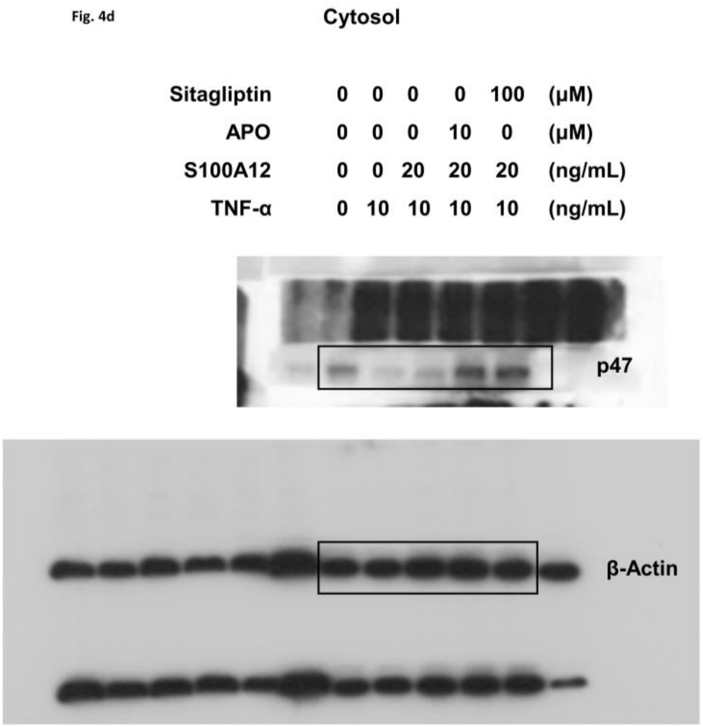

S-Fig. 10. Original full-length gel images of p47 and β-actin in Figure 4d.

Fig. 5a

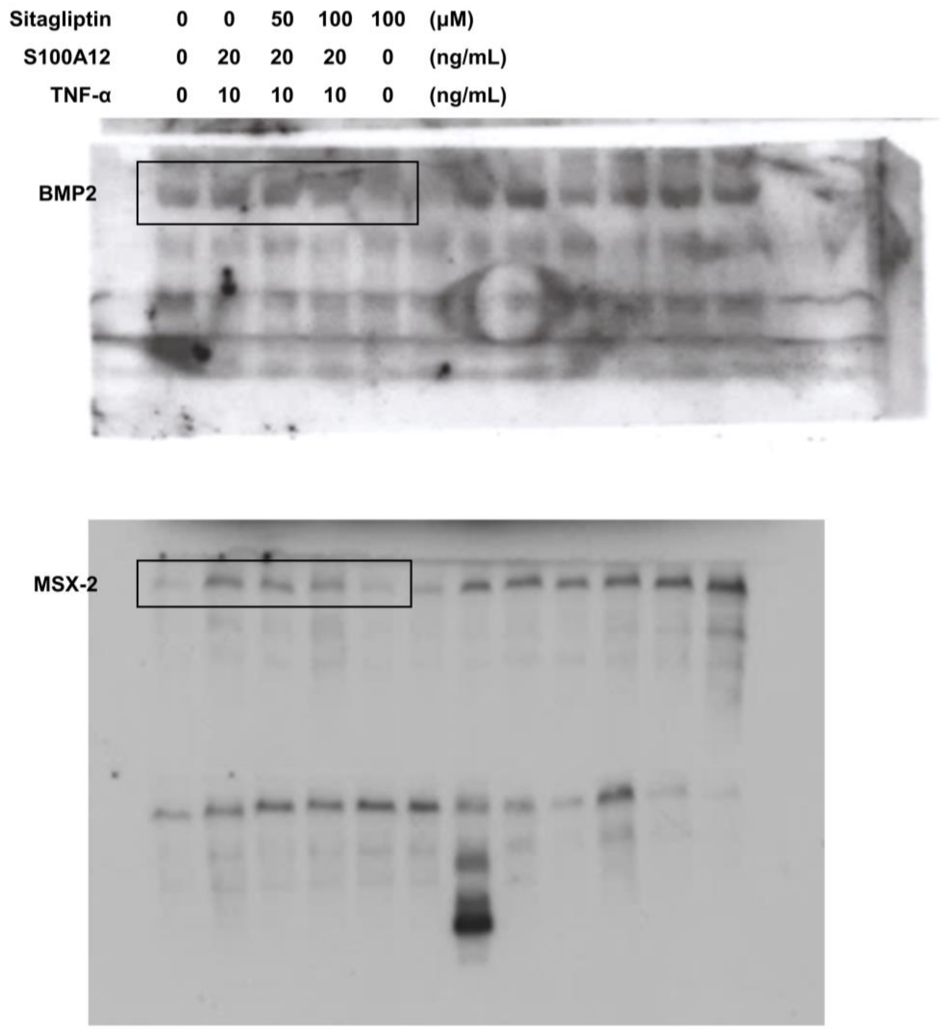

S-Fig. 11. Original full-length gel images of BMP-2 and MSX-2 in Figure 5a.

Fig. 5a

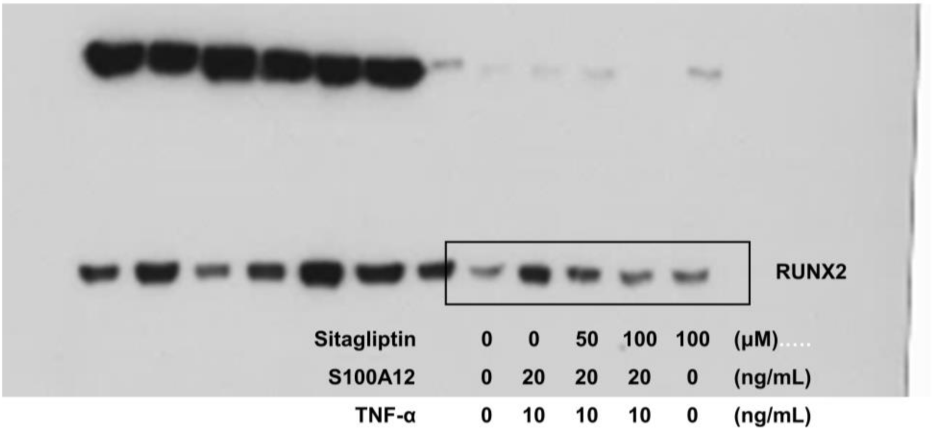

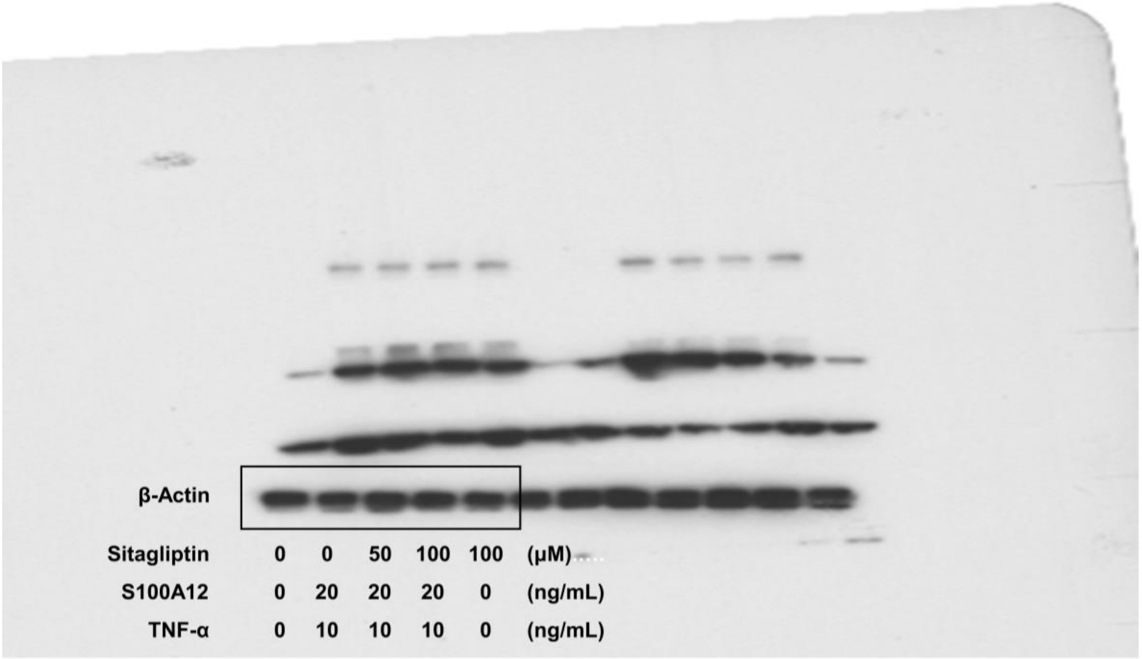

S-Fig. 12. Original full-length gel images of RUNX2 and β-actin in Figure 5a.

Fig. 5c

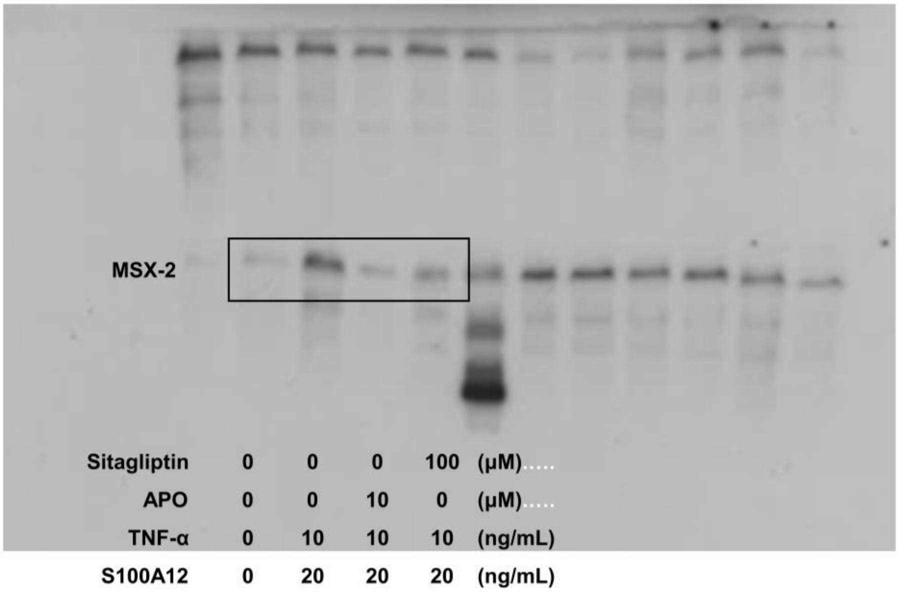

S-Fig. 13. The Original full-length gel image of MSX-2 in Figure 5c.

Fig. 5c

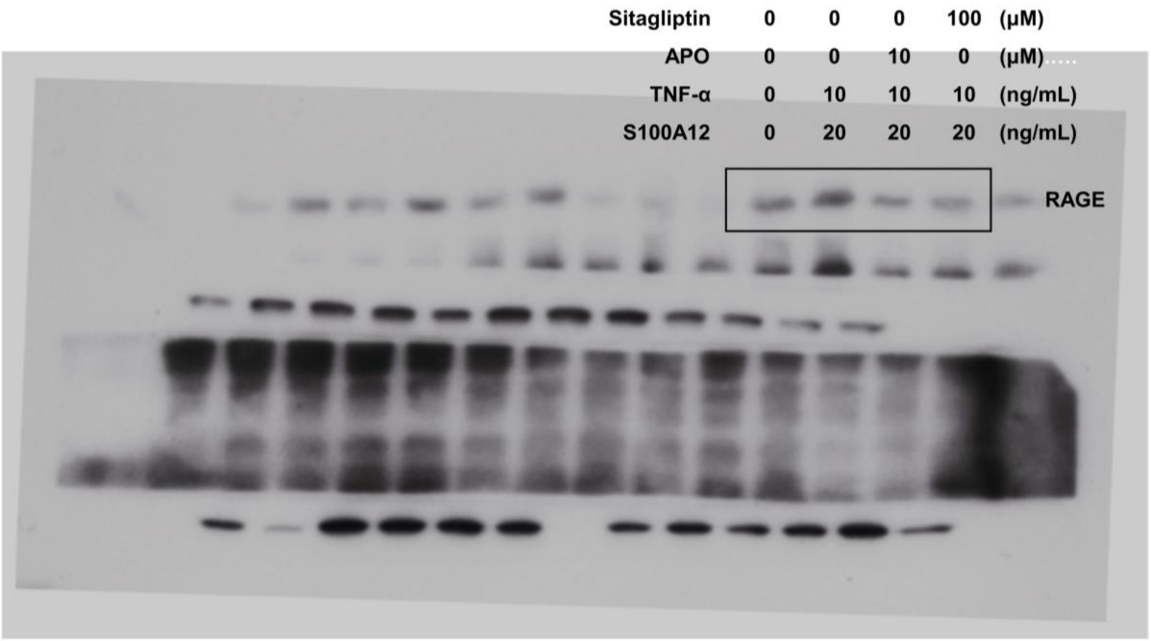

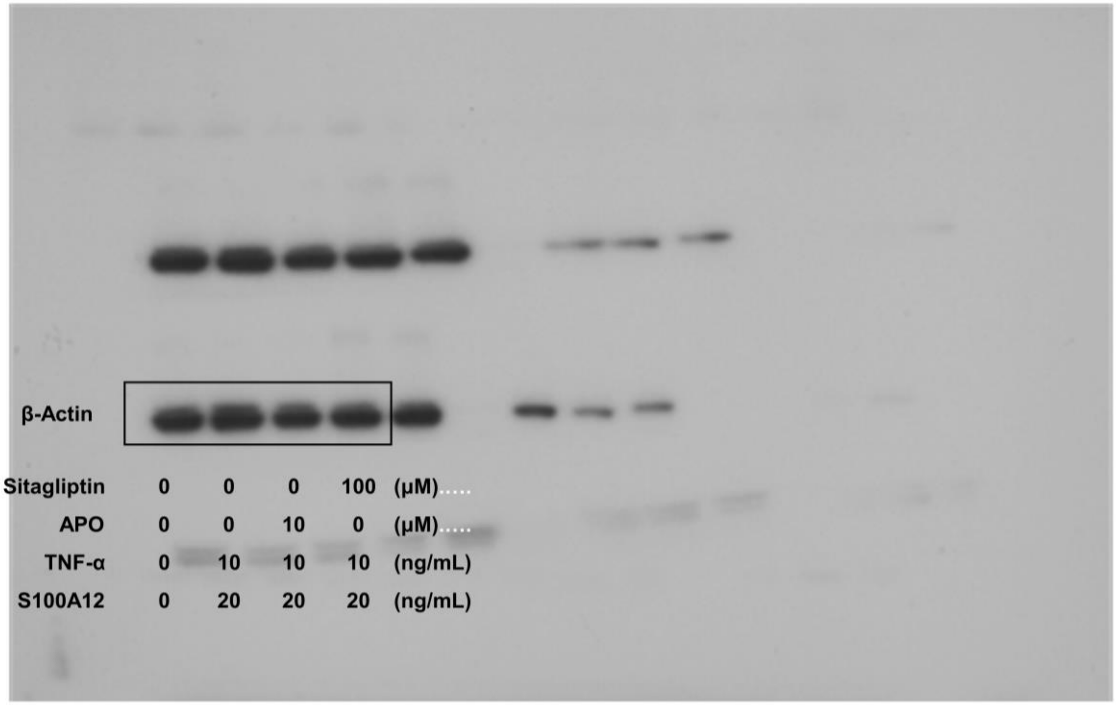

S-Fig. 14. Original full-length gel images of RAGE and β-actin in Figure 5c.
